# Phenotypic Diagnosis of Lineage and Differentiation During Sake Yeast Breeding

**DOI:** 10.1534/g3.117.044099

**Published:** 2017-06-22

**Authors:** Shinsuke Ohnuki, Hiroki Okada, Anne Friedrich, Yoichiro Kanno, Tetsuya Goshima, Hirokazu Hasuda, Masaaki Inahashi, Naoto Okazaki, Hiroyasu Tamura, Ryo Nakamura, Dai Hirata, Hisashi Fukuda, Hitoshi Shimoi, Katsuhiko Kitamoto, Daisuke Watanabe, Joseph Schacherer, Takeshi Akao, Yoshikazu Ohya

**Affiliations:** * Department of Integrated Biosciences, Graduate School of Frontier Sciences, University of Tokyo, Kashiwa, 277-8562, Japan; † Department of Genetics, Genomics and Microbiology, University of Strasbourg, Centre National de la Recherche Scientifique, 67083, France; ‡ Brewing Microbiology Division, National Research Institute of Brewing, Higashi-Hiroshima, 739-0046, Japan; § Research Institute, Ozeki Corporation Ltd., Nishinomiya, 663-8227, Japan; ** Brewing Society of Japan, Kita-ku, 114-0023, Japan; †† Research and Development Department, Asahi Sake Brewing Co., Ltd., Nagaoka, 949-5494, Japan; ‡‡ Department of Molecular Biotechnology, Graduate School of Advanced Sciences of Matter, Hiroshima University, Higashi-Hiroshima, 739-8530, Japan; §§ Department of Biological Chemistry and Food Sciences, Iwate University, Morioka, 020-8550, Japan; *** Pharmaceutical Medical Business Sciences, Nihon Pharmaceutical University, Bunkyo-ku, 113-0034, Japan; ††† Graduate School of Biological Sciences, Nara Institute of Science and Technology, Ikoma, 630-0192, Japan

**Keywords:** single-cell phenotyping, cell morphology, phenotypic diversity, sake yeast

## Abstract

Sake yeast was developed exclusively in Japan. Its diversification during breeding remains largely uncharacterized. To evaluate the breeding processes of the sake lineage, we thoroughly investigated the phenotypes and differentiation of 27 sake yeast strains using high-dimensional, single-cell, morphological phenotyping. Although the genetic diversity of the sake yeast lineage is relatively low, its morphological diversity has expanded substantially compared to that of the *Saccharomyces* *cerevisiae* species as a whole. Evaluation of the different types of breeding processes showed that the generation of hybrids (crossbreeding) has more profound effects on cell morphology than the isolation of mutants (mutation breeding). Analysis of phenotypic robustness revealed that some sake yeast strains are more morphologically heterogeneous, possibly due to impairment of cellular network hubs. This study provides a new perspective for studying yeast breeding genetics and micro-organism breeding strategies.

Over the course of recorded human history, domestication has been used to adapt wild living organisms for human use. Historically, various breeding strategies and selection methods have been used to obtain breeding lines. Genetic theory has been used to breed and produce domesticated organisms, including animals, plants, and micro-organisms. Domestication generally implies a loss of diversity in the species relative to their wild ancestors owing to genetic drift through bottleneck effects (Crow 2010; Amos and Harwood 1998; Bourguiba *et al.* 2012). An understanding of how domestication affects a species provides insights into general mechanisms of adaptation and can guide the genetic improvement of the species through breeding programs. However, it is also true that the ideal organism is not always obtained by breeding, which sometimes results in incomplete or added properties.

Sake is a Japanese alcoholic beverage, which is fermented from steamed rice by the concerted action of a filamentous fungus and yeast (Kanauchi 2013). Amylases secreted from the filamentous fungus, *Aspergillus oryzae*, convert rice starch into glucose. The budding yeast, *Saccharomyces cerevisiae*, then produces ethanol by glycolysis. The concerted action of these two micro-organisms must be maintained during the stepwise fermentation to produce a high concentration of alcohol. In parallel with the ethanol produced by yeast, the production of esters, organic acids, and amino acids is also important for contributing to the aroma and taste (Akiyama 2010). Most sake fermentations are directly inoculated with large amounts of a selected sake yeast isolate to ensure consistent, reliable, and reproducible fermentation. The choice of yeast strain is critical for the aroma and taste characteristics of sake products. Many strategic efforts in yeast breeding have been undertaken to ensure the production of high quality sake (Kitagaki and Kitamoto 2013).

In response to various social demands and individual preferences, sake yeast strains that produce characteristic flavors and tastes have been generated by the Brewing Society of Japan (BSJ) and the National Research Institute of Brewing (NRIB) and distributed by the BSJ. The BSJ and NRIB are an incorporated public interest foundation and a national institute in Japan, respectively, whose precursors were founded in the mid-1900s. Nonfoam-forming sake yeast strains were bred to scale down tank capacity for industrial reasons (Ouchi and Akiyama 1971; Shimoi *et al.* 2002). Advances in fermentation technology allowed the development of ethanol-resistant and high-alcohol-producing sake yeast strains (Hara 1978; Casey and Ingledew 1986; Ogawa *et al.* 2000; Watanabe *et al.* 2011; Shiroma *et al.* 2014). Sake yeast producing high ethyl caproate was isolated to make premium ginjo sake with a fruity flavor and clear taste (Ichikawa *et al.* 1991; Tamura *et al.* 2015). The isolation of mutants (mutation breeding) and the generation of hybrids (crossbreeding) were two major approaches used for breeding.

All sake yeast strains are *S. cerevisiae*. Sake yeast strains were originally named *S. sake* (Yabe-Kozai) (Yabe 1897), *S. yedo*, and *S. tokyo* (Nakazawa 1909); but were later all identified as *S. cerevisiae* (Tsukahara 1962). Genotyping of *S. cerevisiae* indicated that sake yeast strains previously analyzed are diploid, closely related, and very distinct from other populations, such as the laboratory and wine yeast (Schacherer *et al.* 2009; Liti *et al.* 2009; Akao *et al.* 2011; Cromie *et al.* 2013). Although genetic variation within *S. cerevisiae* has been shaped by a complex history influenced by human-associated dispersal and admixture, the genetic variation of sake yeast is typically low, composed of almost purebred yeast.

Breeding processes in general are evaluated by comparing the genotypes and phenotypes between parent and progeny. Expected phenotypic changes that suit the breeding objective directly or indirectly are derived from driver mutations. In addition, unexpected phenotypic changes unrelated to the breeding objective are often observed because of additional off-target mutations or unexpected genetic combinations. It should be noted that yeast morphology was not used for selection during sake breeding (Akiyama 2010). Therefore, morphological changes could be byproducts of breeding. To investigate the process of breeding sake yeast strains, high-dimensional phenotyping (Ohya *et al.* 2015) serves as a powerful tool for monitoring morphological differentiation. Extensive strain-to-strain morphological variations were observed among natural *S. cerevisiae* isolates (Nogami *et al.* 2007; Yvert *et al.* 2013) and protoploid yeast *Lachancea kluyveri* strains (Jung *et al.* 2016). Both studies indicated that morphological variations were not linked to the population structure, geographical origin, or the source environment; suggesting that morphological differentiation occurs more rapidly within the species. This would suggest that rapid and unforeseen phenotypic changes during each step of breeding can be detected by high-dimensional morphological phenotyping. In fact, phenotypic analysis of nonessential genes based on 501 morphological parameters was highly sensitive, with more than two-thirds of the nonessential genes involved in cell morphology (Ohya *et al.* 2005). In addition, cell-to-cell morphological variations (Levy and Siegal 2008) have recently been used to monitor off-target mutations that accumulate during the breeding of a sake strain (Tamura *et al.* 2015).

To gain a better understanding of the lineage and differentiation during sake yeast breeding, we explored single-cell phenomics in sake yeast strains bred by the BSJ and the NRIB. Since the lineage for these strains has been recorded (Akao 2014, 2015), we evaluated every step of breeding using lineage relationships. We characterized intraspecific cellular morphology based on microscopic images of cellular, actin, and nuclear DNA morphology (Ohya *et al.* 2005). After we quantified 501 morphological measurement parameters in 27 sake yeast strains, we obtained >1,500,000 morphological measurements using the CalMorph image processing software. To normalize the morphological values of each trait, we applied a generalized linear model (GLM). Principal component analysis (PCA) was used to reduce the dimension and to generate orthogonal phenotypic space. As a result, this large-scale analysis provided multiple insights into the phenotypic differentiation in terms of morphology among sake yeast strains. Comparison of sake yeast with other natural *S. cerevisiae* isolates revealed that the morphological diversity of sake yeast is substantial. Several general concepts regarding mutation breeding and crossbreeding were uncovered during sake yeast breeding. Cell-to-cell morphological variability was shown to be useful for identifying several yeast strains that were genetically and phenotypically robust to environmental stress (risk-free yeast). Our study provides valuable information on the development of sake yeast, which may also be useful in future studies of breeding strategies for other organisms.

## Materials and Methods

### Strains, culture conditions, fluorescence staining, microscopy, and image processing

Strains used in this study are described in Supplemental Material, Table S1 in File S2. Sake yeast strains and a control strain (BY4743) were cultured at 25° in YPD medium containing 1% (w/v) Bacto yeast extract (BD Biosciences, CA), 2% (w/v) Bacto peptone (BD Biosciences), and 2% (w/v) glucose.

Cells at log phase were fixed and triply stained with FITC-conjugated concanavalin A (FITC-ConA) (Sigma, St. Louis, MO) for the cell wall, rhodamine-phalloidin (rh-ph) (Invitrogen Corp.) for the actin cytoskeleton, and 4’,6-diamidino-2-phenylindole (DAPI) (Sigma) for nuclear DNA, as described previously (Ohya *et al.* 2005). Fluorescent microscopy images of the cells were acquired using an Axio Imager microscope equipped with a 6100 ECplan-Neofluar lens (Carl Zeiss, Germany), a CoolSNAP HQ cooled-CCD camera (Roper Scientific Photometrics, Tucson, AZ), and AxioVision software (Carl Zeiss).

Microscopy images of the cells were analyzed with CalMorph (ver. 1.3) image processing software designed for diploids (Yvert *et al.* 2013). We obtained the morphological data of the 501 traits from the single-cell data. Descriptions of each trait were presented previously (Ohya *et al.* 2005). The CalMorph user manual is available at the *Saccharomyces cerevisiae* Morphological Database (SCMD; http://yeast.gi.k.u-tokyo.ac.jp/datamine/).

### GLM and statistical tests

We detected morphological differences between the sake yeast strains and the standard strain and normalized the morphological values in each trait using a GLM. Probability density functions (PDFs) and accompanying link functions in the GLM were assigned to each trait as described previously (Yang *et al.* 2014). Maximum likelihood estimation and the Wald test were performed using the gamlss and coeftest functions in the gamlss (Stasinopoulos and Rigby 2007) and lmtest (Zeileis and Hothorn 2002) R packages, respectively. All statistical analyses were performed using R (https://cran.r-project.org/).

To statistically test the morphological differences between sake yeasts and natural yeast isolates, and between nonfoam-forming (*awa*) mutants and their parent strains, we conducted two-way ANOVA of the GLM without an interaction term for each trait. For sake and natural yeast isolates, we used the morphological data of 27 sake yeast strains (*n* = 5) cultured in YPD medium, 36 natural yeast strains (*n* = 5) cultured in SD medium, and 45 replicates of BY4743 cultured in YPD (*n* = 11) and in SD (*n* = 34) to compare the morphological phenotypes between sake and natural yeast isolates. The morphological data of 36 natural yeast isolates (*n* = 5) and BY4743 (*n* = 34) were acquired previously (Yvert *et al.* 2013). Categories of the strains and the media were incorporated as explanatory variables in the linear model. Representative values of each strain, effects of medium, and dispersion values of experimental error in each trait were estimated with the linear model by maximum likelihood estimation. *Z* values of each strain from BY4743 in all traits were estimated using the Wald test, after subtracting the effects of the medium with dispersion values, and used for PCA. For PCA in Figure 2 and Figure 3, a single *Z* value was calculated for each strain in each trait from replicated values in each strain. For PCA in Figure 6 and Figure 7, the *Z* value was calculated for each replicated value in each strain. For *awa* mutants and their parents, we employed morphological data of six *awa* mutants (K601, K701, K901, K1001, K1401, and K1501) and five parental strains (K6, K7, K9, K10, and K14). Categories of the strains and the *awa* mutants were incorporated as the explanatory variables into the linear model. We selected 56 of the 501 traits to have the minimum Akaike information criterion (AIC) among the other models with or without the explanatory variables of the strains, the medium, and/or the interaction term among them. Then, 21 out of the 56 traits were fitted to the selected model by the likelihood ratio test at *P* < 0.05 after Bonferroni correction. Finally, of the 21 traits, seven (Figure S4 in File S1) were detected to have significant effects on the *awa* mutants at *P* < 0.05 after the Bonferroni correction by the Wald test.

To statistically test the morphological differences among the sake yeast strains, and between K1001 and K1701 strains, we conducted one-way ANOVA of the GLM for each trait. Categories of the strains were incorporated as the explanatory variable into the linear model. For the sake yeast strains, we employed morphological data of 27 sake yeast strains (*n* = 5) and 11 replicates of BY4743 cultured in YPD. *Z* values of each sake strain from BY4743 in all traits were estimated using the Wald test and used for PCA. For the K1001 and K1701 strains, we employed five replicated morphological data sets for each strain. Applying one-way ANOVA between K1001 and K1701 to all 501 traits, 26 of the 501 traits were found to differ significantly at *P* < 0.05 after the Bonferroni correction by the likelihood ratio test (Table S4 in File S2). Of the 26 traits, 14 were grouped by PCA for the *Z* values of 34 replicates of BY4743, as described previously (Ohnuki *et al.* 2012).

To statistically test mean values of cell size at S/G2 (Figure 5A) and the ratio of unbudded cells (Figure 5B), the mean of five replicated values was estimated in each strain using a GLM as described above. We then performed Wilcoxon–Mann–Whitney *U*-test between K7 and old sake yeast groups using the wilcox.test function of R in the default package.

The phenotypic potentials (PPs) (Figure 8A) of 27 sake yeast strains and BY4743 were calculated as previously described (Yvert *et al.* 2013). The difference between BY4743 and each sake strain was tested by Dunnett’s test at *P* < 0.05 using the glht function in multcomp of the R package (Hothorn *et al.* 2008).

### PCA for reducing dimension and generating orthogonal space

PCA in Figure 2 was applied to the *Z* values of each strain for all 501 traits that were calculated, using a GLM as mentioned above. From the PCA for 27 sake yeasts, 36 natural yeasts, and BY4743, the cumulative contribution ratio (CCR) of the first 7, 11, 16, and 28 PCs reached 60, 70, 80, and 90%, respectively (Figure S1 in File S1). Similarly, from the PCA for the mean *Z* values of 27 sake yeasts and BY4743 in Figure 6 and Figure 7, the CCR of the first 6, 8, 11, and 16 PCs also reached 60, 70, 80, and 90%, respectively (Figure S2 in File S1). Morphological features of each PC axis in Tables S2 and S3 in File S2 were extracted by successive PCA for *Z* values of 114 replicates of BY4743 as described previously (Ohnuki *et al.* 2014).

### Euclidean distance in the degenerated morphological space

The Euclidean distance (Deza and Deza 2009) was used to assess the morphological differences between two yeast strains. The Euclidean distance *d* between strain *x* and strain *y* was calculated by the following equation:

$$d\left(x,y\right)=\sqrt{\sum {}_{i=1}^{N}{\left(x_{i}-y_{i}\right)}^{2}},$$

where *x_i_* and *y_i_* were the *i*th *Z* values (*i* = 1, 2, 3, …, *N*) of independent morphological traits after degenerating the 501 morphological traits into an *N*-dimensional phenotype. The Euclidean distance *d* is close to zero if the cell morphology of the two strains is similar; otherwise, *d* increases.

The Euclidean distance between each strain and its parent in Figure 6 and Figure 7 was calculated from the PC scores of the first 16 PCs (CCR 90%). To calculate the PC scores of each strain, *Z* values from each independent experiment in each strain were projected onto the 16 PCs. The Euclidean distance between two arbitrary strains was calculated for all possible pairs among the replicated values in each strain (*e.g.*, the Euclidean distance of 25 combinations was calculated from five replications between two strains). Similarly, the Euclidean distance in each cross was calculated from the distance between the hybrid and the middle point of the parents. The middle point of the parents was calculated for all possible pairs of replicated values between the parent strains (*e.g.*, 25 middle points were calculated from five replications between parent strains). Subsequently, the Euclidean distance was calculated for all possible pairs between the hybrid and the middle points (*e.g.*, the Euclidean distance of 125 combinations was calculated from five replications of the hybrids and 25 middle points of the parents). The mean value and the SD were calculated from the Euclidean distance of all possible pairs. The ratio of the Euclidean distance in Figure 7D was a normalized Euclidean distance independent of the distance of their parents, and calculated as the Euclidean distance in each cross divided by the distance between their parents. PC scores of the 62 segregants between BY and RM were calculated as previously described (Yang *et al.* 2014). The ratio of Euclidean distance of the 62 segregants was calculated from the 33 PCs which reached 90% of the CCR. The ratio of the Euclidean distance from the middle point of the parents to the hybrids was compared between the 62 segregants and the sake yeasts (K13, K1601, and K1801) by Wilcoxon–Mann–Whitney *U*-test at *P* < 0.01 after the Bonferroni correction.

### Hierarchal cluster analysis

Hierarchal cluster analysis (HCA) in Figure 3C and Figure S3 in File S1 was performed using the hclust function in the default package of R. Dissimilarity was calculated by subtracting the correlation coefficient *R* from one, where *R* was calculated from the PC scores of six PCs (60% CCR) from PCA for 27 sake strains and BY4743. The dendrogram in Figure 3C was generated from the average linkage of the dissimilarity using the plot.phylo function in ape of the R package (Paradis *et al.* 2004). The significance of each cluster in Figure S3 in File S1 was estimated from the approximately unbiased probability value (AU *P*-value) using the pvclust function in the pvclust package of R (Suzuki and Shimodaira 2006).

### Neighbor-joining tree building

The biallelic segregating sites detected among a set of 36 *S. cerevisiae* strains representative of the species diversity (Strope *et al.* 2015) and 24 sake isolates from the 1002 Yeast Genomes Project (http://1002genomes.u-strasbg.fr) were used to elucidate the phylogenetic relationships among them. Based on these sites, a distance matrix was first computed and submitted to bionj algorithm (R packages ape and SNPrelate) to construct a neighbor-joining tree.

### Drug sensitivity

After preculture in YPD medium at 25° for 12 h, the number of yeast cells was adjusted to 1.0 × 10^7^ cells/ml. The cell suspension was diluted serially and inoculated onto YPD plates, each containing one of the following: 10 μg/ml benomyl (Sigma-Aldrich), 100 mM HU (Sigma-Aldrich), or 50 μg/ml calcofluor white (Sigma-Aldrich). After incubation at 25° for 5 d, the cell growth (drug sensitivity) on the plate was observed. Three homozygous gene deletion strains (*rad52*Δ, *pac10*Δ, or *fks1*Δ) from a gene knockout collection (EUROSCARF) were used for drug sensitivity controls.

### Data availability

All morphological data used in this study are available at http://www.yeast.ib.k.u-tokyo.ac.jp/sakeyeasts.

## Results

### High-dimensional morphological phenotyping of sake strains

**Figure 1:**
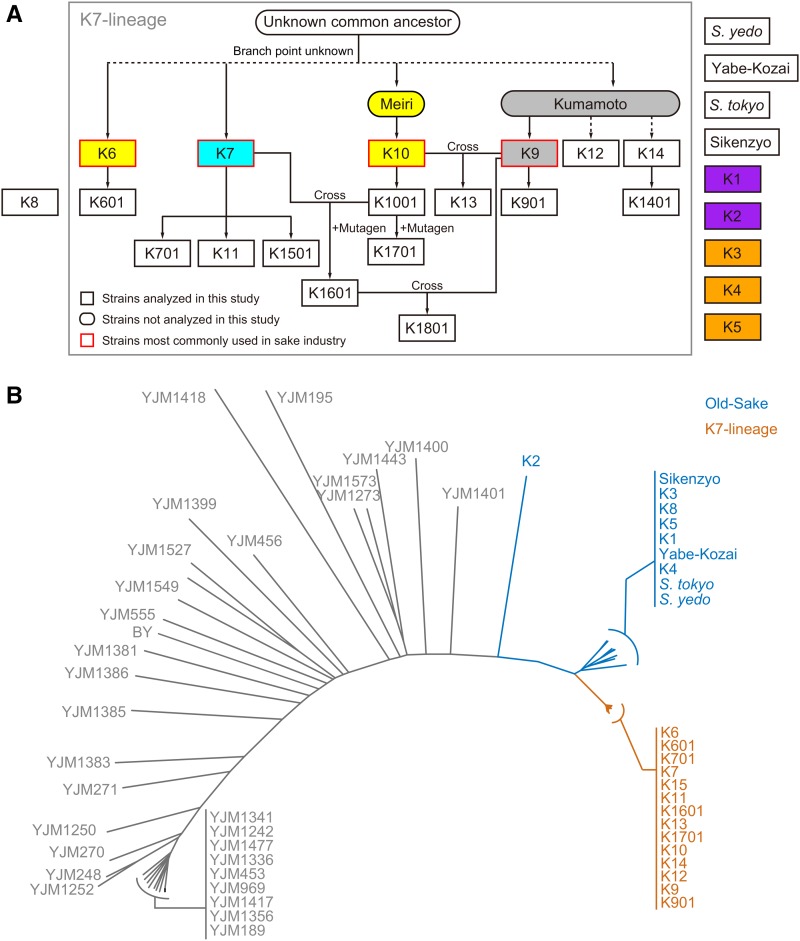
Tree diagram of sake yeast lineage and breeding. (A) Tree diagram of sake yeast strains. Rectangles and ovals indicate sake yeast strains analyzed or not analyzed in this study, respectively. Strains in the gray box (K7 lineages, also referred to as the K7 group previously) constitute the group of sake yeasts that putatively share a common ancestral origin. Among the K7 lineages, the four strains highlighted in red (K6, K7, K9, and K10) are the strains used as the original breeding strains. Symbol colors indicate the geographical origin of the strains; yellow, blue, violet, orange, and gray denote the Tohoku, Chubu, Kinki, Chugoku, and Kyushu regions in Japan, respectively (Table S1 in File S2). Arrows between strains indicate breeding history (*e.g.*, K601 is isolated from K6), and if isolation was conducted by the crossing of two parental strains (Cross) and/or mutagen treatment (+Mutagen), both are indicated beside them. The diagram was constructed based on Japanese literature (Akao 2014, 2015). (B) Neighbor-joining tree of natural *S. cerevisiae* strains (gray) representative of the species diversity (Strope *et al.* 2015) and the sake yeast strains (K7-lineage strains in orange and old sake yeast strains in blue, the 1002 Yeast Genomes Project). Sake yeast strains are distinct from the rest of the population and distributed in two clusters that are in accordance with their associated lineages.

To comprehensively understand morphological differentiation during sake yeast breeding, we performed high-dimensional, morphological phenotyping of all sake yeast strains distributed by the BSJ, which are stored at the NRIB in Japan and were recently used for genotyping studies (Table S1 in File S2). The sake strains that are currently used and distributed by the BSJ are all Kyokai no. 7 (hereafter called K7) lineage strains that were derived from four established strains (K6, K7, K9, and K10; Figure 1A, red rectangles). In addition to the currently used sake strains, we analyzed K8 and nine other sake yeast strains which were used previously but are no longer distributed for sake brewing and are therefore designated as “old sake yeast” hereafter (Figure 1A, to the right of the gray box). K7-lineage strains and old sake yeast strains are distinct in origin, but restricted to a limited portion of the *S. cerevisiae* phylogenetic tree (Figure 1B). The sake yeast strains exhibited predominantly unattached individual cells in culture, making it possible to perform a semiautomated image analysis of single cells using CalMorph (Ohya *et al.* 2005). We cultured cells of each strain in a rich medium as five biological replicates; fixed cells with formaldehyde; and stained the cell wall, nuclear DNA, and actin with FITC-ConA, DAPI, and rh-ph, respectively. Images of at least 200 cells per culture were acquired using fluorescent microscopy and analyzed with CalMorph to quantify 501 traits reflecting cell size, shape, orientation, and intracellular organization. Altogether, morphological data of >1000 cells were acquired for each strain, allowing the statistical analysis of morphological differences among the sake strains.

### Morphological diversity of sake yeast strains

**Figure 2:**
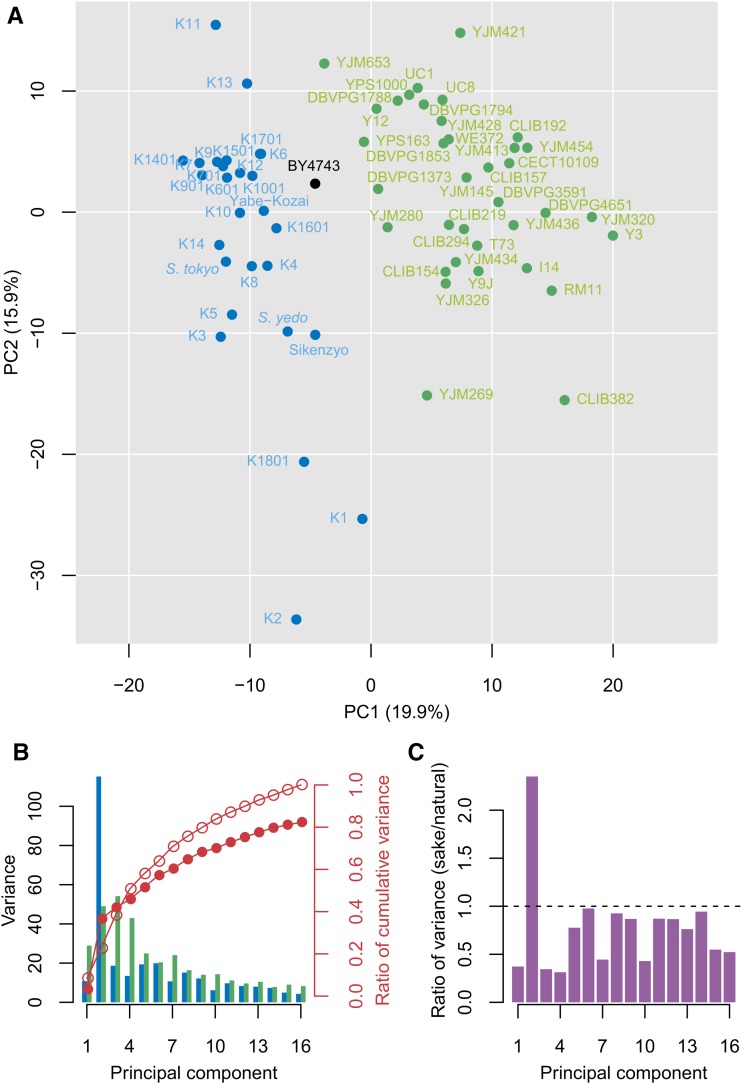
Comparison of phenotype variation between sake and natural yeast strains. (A) Distribution of morphological phenotypes among sake and natural yeast strains for PC1 and PC2. Blue, green, and black circles indicate sake, natural, and BY4743 yeast strains, respectively. Percentages in parentheses on each axis indicate the contribution ratio. (B) Phenotypic variance of sake and natural yeast strains for each PC. Blue and green bars (left axis) indicate variance of sake and natural yeast strains, respectively. Red circles (right axis) indicate the ratio of cumulative variance of the sake yeast strains (filled circles) and the natural yeast strain (open circles) to the sum of variances in the 16 PCs (PC1–PC16) of the natural yeast strains. Cumulative proportion of variance from PC1 to PC16 explained 80% of total variance. (C) Ratio of variance of sake yeast strains to that of natural yeast strains for each PC. Horizontal dashed line indicates point of equal variance between sake and natural yeast strains.

To understand the morphological diversity of the sake yeast strains, we compared the morphological variation of sake yeast strains with that of natural *S. cerevisiae* isolates. To make this comparison, we first identified morphological differences of the yeast strains from the standard strain (BY4743) for each parameter and normalized the morphological value by applying a GLM as described previously (Yang *et al.* 2014). We used the high-dimensional morphological data of 36 typical natural yeast isolates previously published (Yvert *et al.* 2013) as a reference. This set of isolates was selected from various geographical and ecological origins. These strains belong to a panel that was previously used to explore the genetic diversity of the species (Schacherer *et al.* 2009). Morphological variations between the sake yeast strains and the 36 natural yeast isolates were then examined by focusing on the diverse expansion of the population in the degenerated orthogonal phenotypic space (Figure 2A). This was achieved by performing PCA with the sake yeast data and the reference data combined, as described previously (Yang *et al.* 2014). An advantage of comparing in the degenerated orthogonal space is that one can exclude bias caused by the correlation between the morphological parameters. We found that variation in the sake yeast strains (Figure 2A, blue dots) was comparable to that of the natural yeast strains (Figure 2A, green dots) in the phenotypic space comprised of principal component 1 (PC1) and PC2. The ratios of variance of sake yeast strains to natural yeast strains in PC1 and PC2 were 0.37 and 2.4, respectively. Since the contributions of PC1 and PC2 only accounted for 20 and 16% of the variance, respectively, we also checked the variation of the two populations until the cumulative contribution reached 80% (Figure S1 in File S1, first 16 PCs) and found that the ratio of cumulative variance of sake yeast strains to the natural yeast isolates finally reached 82% (Figure 2B). Analyses of the ratio of variance in the first 16 principal components revealed relatively higher ratios in PC2 and PC6, corresponding to cell size and nuclear localization noise in the mother cell, respectively (Figure 2C and Table S2 in File S2); indicating that some of the morphological features were quite variable among the sake yeast strains. In fact, the genetic diversity of the sake subpopulation (π = 0.9 × 10^−3^) is twofold lower than the one found in the set of 36 natural phenotyped isolates (π = 1.9 × 10^−3^) (Schacherer *et al.* 2009; Yvert *et al.* 2013). Interestingly, the low genetic diversity stands in contrast to the substantial morphological diversity among sake yeast strains revealed by our data. We also noticed that the two populations were almost separated in the degenerated orthogonal phenotypic space (Figure 2A). The major difference between natural and sake yeast strains was associated with PC1, relating to nuclear size and cell-to-cell variation in some morphological features (Table S2 in File S2). This suggests that sake yeast exhibit distinct morphological features compared with other natural *S. cerevisiae* isolates. Sake yeast strains isolated in the early days (*S. sake*, *S. Tokyo*, and *S. yedo*) and *S. cerevisiae* were originally classified into different species (Yabe 1897; Nakazawa 1909). This may reflect the fact that these sake yeast strains have distinct morphology.

### Morphological traits of sake yeast differentiated by geographical origin and period of distribution

**Figure 3:**
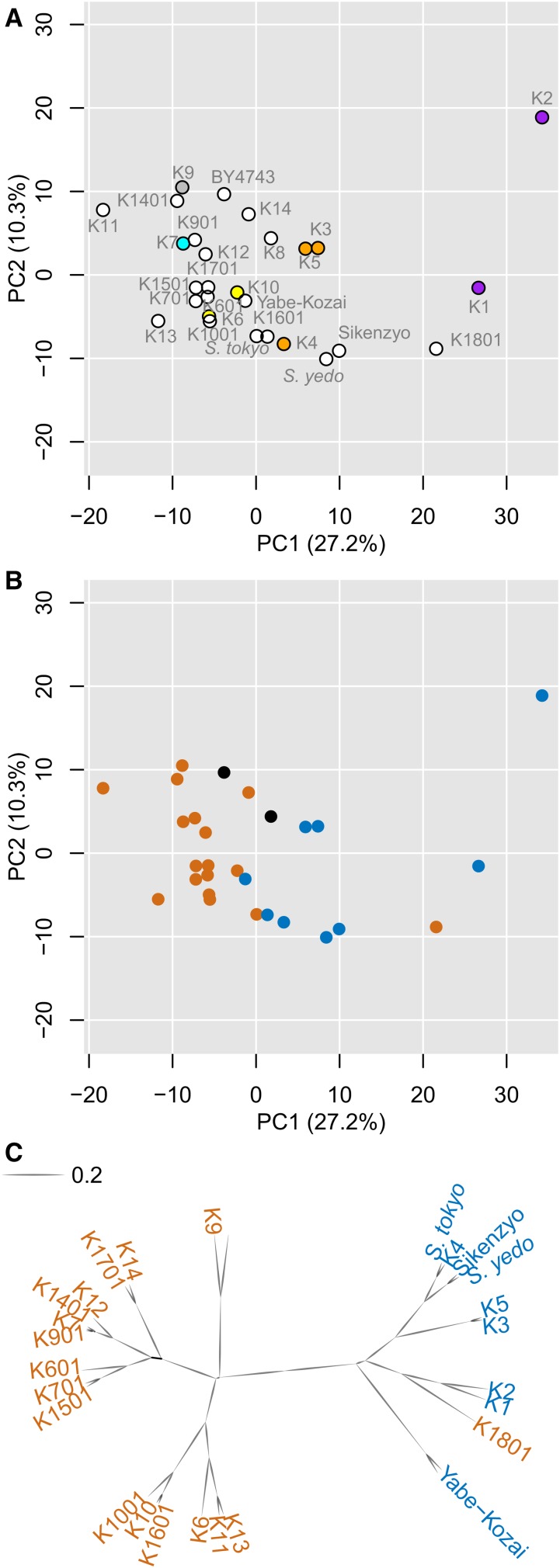
Phenotypic variation of sake yeast strains. (A) Phenotype distribution of sake yeast strains. The color legends of the circles are the same as in Figure 1A; yellow, blue, violet, orange, and gray denote the Tohoku, Chubu, Kinki, Chugoku, and Kyushu regions in Japan, respectively. Percentages in parentheses on each axis indicate the contribution ratio. (B) Phenotypic distribution of K7-lineage strains, old sake yeast strains, and BY4743. Orange, blue, and black circles indicate K7-lineage, old sake yeast, and other strains, respectively. Percentages in parentheses on each axis indicate the contribution ratio. (C) A morphological cluster dendrogram of 27 sake yeast strains and BY4743. The dendrogram was generated based on morphological similarity computed by the correlation coefficient of PC scores of 6 PCs (CCR = 60%). Scale bar indicates the mean of correlation coefficient. Orange and blue indicate K7-lineage and old sake yeast strains, respectively.

**Figure 4:**
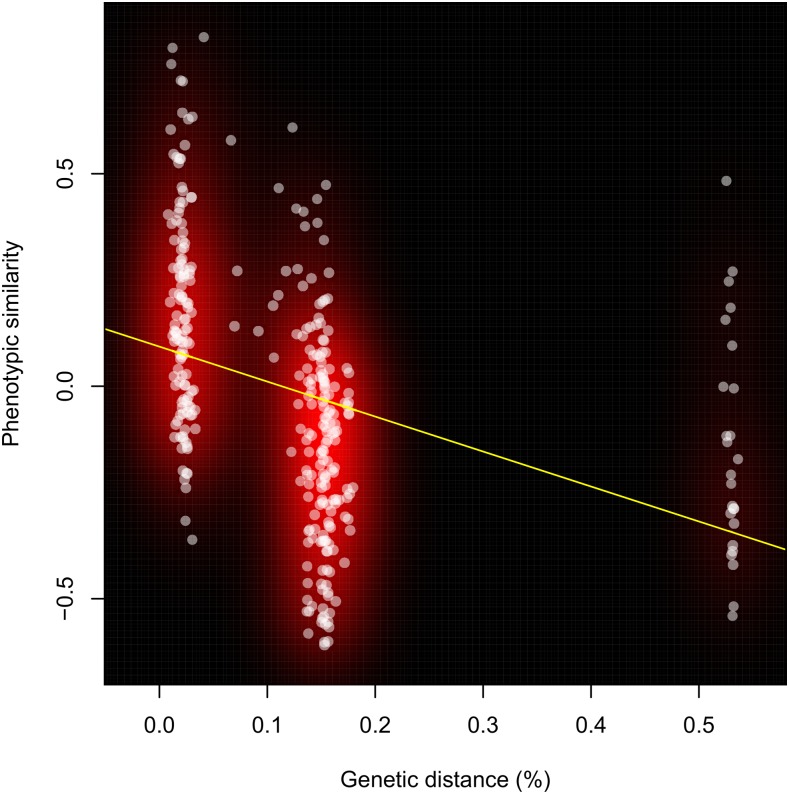
Correlation between genetic diversity and trait profile variation. Pairwise genetic diversity between sake yeast strains (the 1002 Yeast Genomes Project) was computed by the proportion of SNP differences using the S288c genome sequence as reference genome. The resulted value is plotted against phenotypic similarity computed using the correlation coefficient of PC scores of 16 PCs (CCR = 90%) in PCA for cell morphology. All 325 possible pairs of 26 distinct sake strains (27 sake strains except the K1801 strain) are represented as white dots. The yellow line corresponds to the linear regression of genetic distance to phenotypic similarity (|R| = 0.37, Kendall’s τ rank correlation test, *P* = 2.4 × 10^−23^). Discrete distribution of genetic distance values reflects the phylogenetic relationship of the tested sake yeast strains: K7-lineage strains, old sake strains (other than K2), and K2. The density of dots was estimated by kernel density estimation with Gaussian distribution and is highlighted in black to red colors.

To determine whether morphological features are specific to geographical origin, we mapped the sake strains in their own degenerated orthogonal phenotypic space (Figure 3A). For this purpose, we performed PCA only with the sake yeast data set to capture the most prominent intraspecies morphological variations. The first two components, PC1 and PC2, were explained by 38% of the variance in total and correlated with cell size and cell elongation, respectively (Figure S2 in File S1 and Table S3 in File S2). Sake strains from Kyushu (gray dot) and Tohoku (yellow dots) were plotted with a low PC1 score, while those from Kansai (violet dots) were plotted with a high PC1 score (Figure 3A). Sake strains from Kyushu and Tohoku were all assigned to the K7 lineage, while sake strains from Kansai were distributed by the Meiji era, and were classified as old sake yeast. Therefore, we next differentiated the currently-used K7-lineage sake strains (orange dots) and old sake yeast strains (blue dots) in the degenerated space (Figure 3B), and found that these two populations were nearly separated. Clustering analysis was performed based on the phenotypic distance between the sake yeast strains (Figures 3C and Figure S3 in File S1), showing that the K7-lineage sake strains and old sake yeast strains clustered almost completely into different groups. We then tried to directly determine the relationship between genetic distance and morphological distance (see *Materials and Methods*). There were three groups with a genetic distance of 0.01, 0.15, and 0.5, showing the relationship between genetic distance and morphological distance (Figure 4). This suggested that genetic diversity influences morphological profiles in the sake yeast strains.

**Figure 5:**
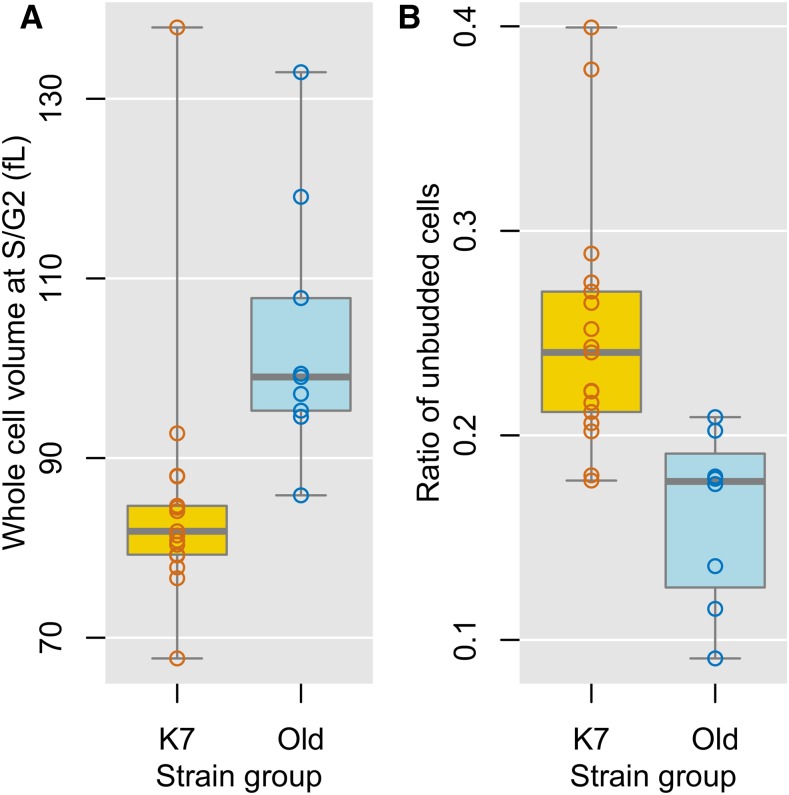
Morphological differences between K7-lineage strain and old sake yeast strains. (A) Whole cell size at S/G2. The whole cell volume at S/G2 is calculated in femtoliter assuming the volume scales like the three-halves power of the area. Circles indicate mean values of each strain (*n* = 5). (B) Ratio of unbudded cells. All old sake yeast strains except for K2 are plotted. Circles indicate mean values of each strain (*n* = 5).

The old sake yeast strains had high PC1 scores (Figure 3B), which correlated with large cell size. We therefore compared their sizes, and found that on average, the old sake yeast strains were significantly larger (21%) than the K7-lineage strains (Wilcoxon–Mann–Whitney *U*-test, *P* < 0.05; Figure 5A). These results suggest that the old sake yeast strains are larger, possibly due to shared ancestral mutations.

A previous morphological study showed that the laboratory strain (X2180) is larger than the K7-lineage strains, K6, K7, K9, and K10 (Watanabe *et al.* 2011). This increased size in the laboratory strain is due to a lower expression level of the G1 cyclin gene, *CLN3* (Watanabe *et al.* 2011). Lower *CLN3* expression levels also result in an accumulation of unbudded cells. If the larger cell size of the old sake yeast strains was also due to lower *CLN3* expression, these strains would also show a higher proportion of unbudded cells. However, contrary to our expectations, these strains showed a significant accumulation of budded cells (Wilcoxon–Mann–Whitney *U*-test, *P* < 0.05, Figure 5B), suggesting that the lower *CLN3* expression level is not the reason for the larger cell phenotype in the old sake yeast strains.

### Morphological change upon mutation breeding

One of the breeding strategies used for sake yeast is mutant isolation. After spontaneous mutations arise or artificial mutations are introduced with mutagens into the parental strain, yeast cells are selected under several conditions and evaluated rigorously for the desired phenotype. Many sake yeast strains have been developed by the BSJ and the NRIB using this method, including the ethanol-resistant sake yeast (K11), nonfoam-forming sake yeasts (K601, K701, K901, K1001, K1401, and K1501), and sake yeasts which produce certain flavors (K12 and K14).

**Figure 6:**
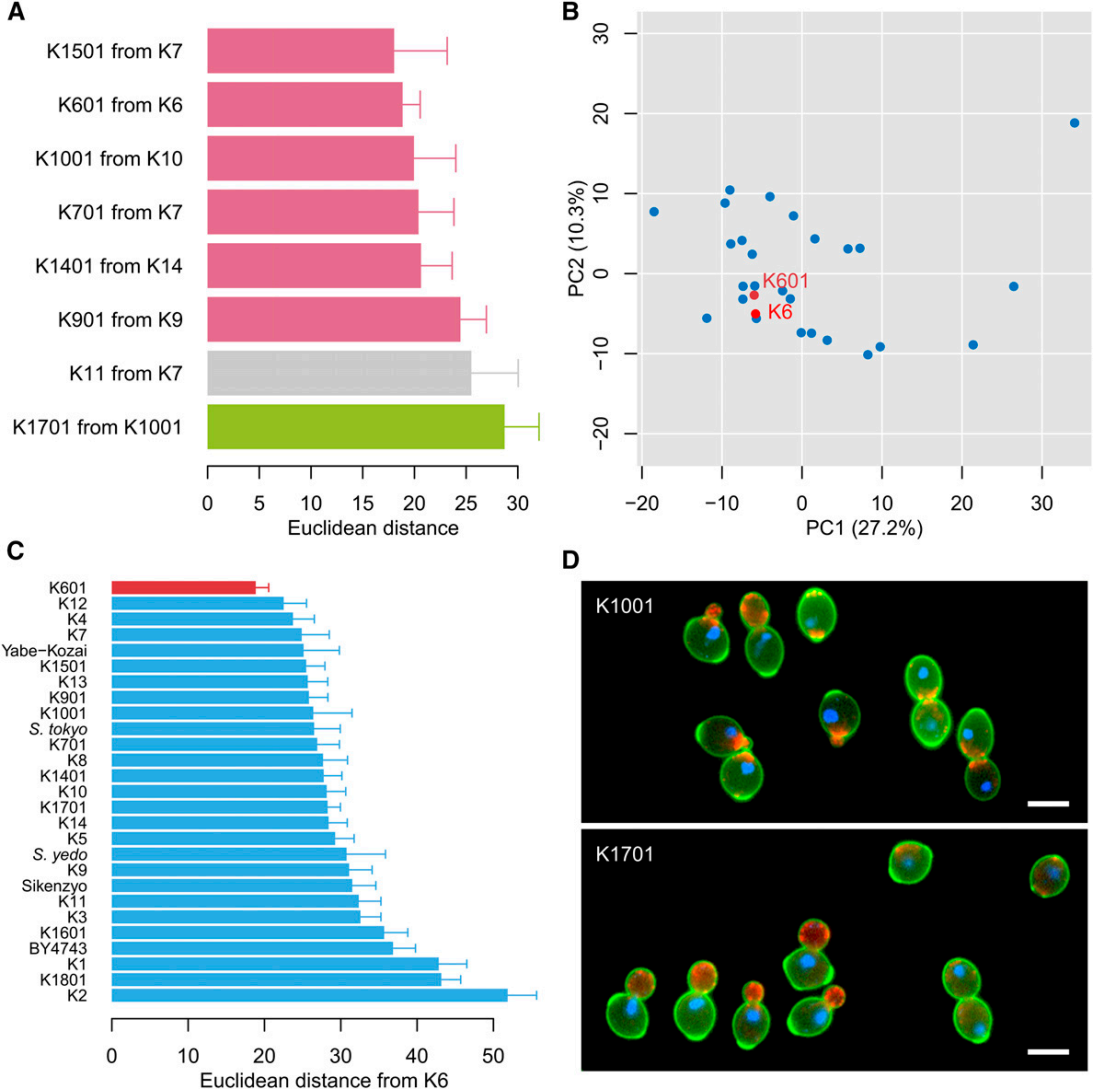
Morphological differentiation upon mutation breeding. (A) Distribution of Euclidean distance from parent strain. The Euclidean distance was calculated from PC scores of 16 PCs (CCR = 90%). The green bar indicates a sake yeast strain isolated with mutagen. Pink bars indicate nonfoam-forming sake yeast. Error bars indicate SD. (B) Distribution of phenotypes between parent strain and its progeny. Red circles indicate the parent strain (K6: bright red) and its progeny (K601: dark red). (C) Distribution of Euclidean distance from K6. The Euclidean distance was calculated from PC scores of 16 PCs (CCR = 90%).The color legends are the same as in (B). Error bars indicate SD. (D) Photographs of K1001 and K1701 strains. Green, red, and blue represent cell shape, actin, and nuclear DNA, respectively. Bar, 5 μm.

We evaluated the morphological lineage during breeding based on the lineage relationships among these sake yeast strains (Figure 1A). The degree of morphological change during breeding was calculated using the Euclidean distance (see *Materials and Methods*) in every parent-and-progeny pair of sake yeast strains. We found that fewer morphological changes were observed during the breeding of nonfoam-forming sake yeast (Figure 6A, pink bars). Parental foaming strains reportedly express sake yeast-specific *Awa1p* on the surface of the cell wall, which is hydrophobic and adheres to carbon dioxide gas bubbles to form a stable foam (Shimoi *et al.* 2002; Ouchi and Nunokawa 1973). In contrast, the developed nonfoam-forming sake yeast has defects in *AWA1*, becoming hydrophilic and easily mixing with water while ignoring gas bubbles (Shimoi *et al.* 2002). As the six nonfoam-forming strains gained common morphological changes during breeding, with statistically altered values in seven morphological parameters (Wald test, *P* < 0.05 after the Bonferroni correction; Figure S4 in File S1), these morphological changes were likely caused by the *awa1* lesion. We next examined positional relationships between parent and progeny in the degenerated orthogonal morphological space and found that one of the nonfoam-forming mutants, K601, which is derived from K6, lay adjacent to K6 (Figure 6B). We compared the morphology of K6 with those of all sake yeast strains and found that K601 was the most similar to K6 among the 26 strains examined (Figure 6C), confirming the morphological similarity between the parent (K6) and its progeny (K601).

We also compared the morphologies of spontaneous mutants and a mutagenized strain, K1701. Calculation of the Euclidean distance between parent and progeny revealed that the morphology of the mutagenized strain K1701 was substantially altered. The morphology of mutagenized strain K1701 was the most changed among the strains developed by mutation breeding (Figure 6A, green bar). Multiple differences were observed in four distinct morphological features (cell size, polarized actin patch localization, actin region size, and cell cycle stages of actin patch localization) related to cell shape and actin patch morphology (Figure 6D, Figure S5 in File S1, and Table S4 in File S2). Most of these extensive morphological changes were possibly unexpected phenotypes, unrelated to the fermentation properties of K1701. This suggests that mutation breeding with mutagens caused the accumulation of off-target mutations, resulting in substantial morphological alterations.

### Morphological change upon crossbreeding

**Figure 7:**
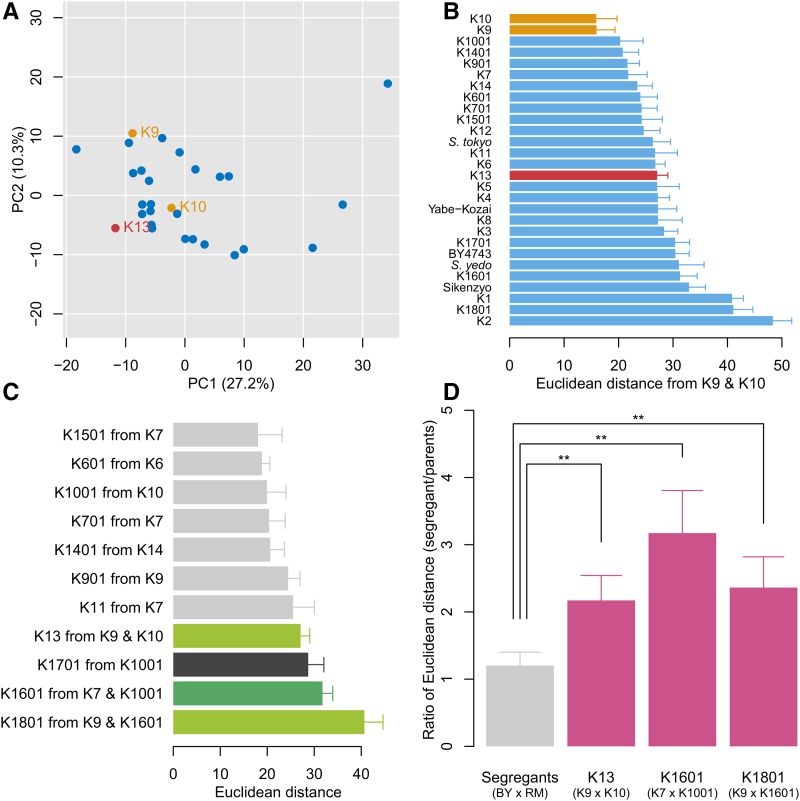
Morphological differentiation upon crossbreeding. (A) Distribution of phenotypes between parent strains and the hybrid strain. Orange and red circles indicate the parent strains (K9 and K10) and the hybrid strain (K13), respectively. (B) Distribution of Euclidean distance from the middle point between K9 and K10. The Euclidean distance was calculated from PC scores of 16 PCs (CCR = 90%). The color legends are the same as in (A). Error bars indicate SD. (C) Distribution of Euclidean distance from parent strains. The Euclidean distance was calculated from PC scores of 16 PCs (CCR = 90%). Dark and light green bars indicate hybrids generated with and without mutagens, respectively. Black and gray bars indicate other isolated strains with and without mutagens, respectively. Error bars indicate SD. (D) Morphological diversity of the segregants. Red and gray bars indicate the sake yeast strains and segregants from two distantly related yeast strains, BY and RM, respectively. The ratio of Euclidean distance (*y*-axis) was calculated by dividing the distance from the middle point of the parents to the progeny by that to the parents. Euclidean distance was calculated from PC scores that reached to 90% of CCR. Error bar indicates SD. ** *P* < 0.01 after Bonferroni correction by Wilcoxon–Mann–Whitney *U*-test (*n* = 62, 100, 125, and 125 in the segregants from BY × RM, K13, K1601, and K1801, respectively).

Crossbreeding is the other breeding strategy used for sake yeast. Hybrid diploid strains can be bred after mating two haploid strains with different mating types derived from the parental sake yeast diploid strains. Ester-producing sake yeast strains (K13, K1601, and K1801) have been developed by the BSJ and NRIB using crossbreeding. We first compared the morphology of K13 with that of its parent strains, K9 and K10. Examination of the degenerated orthogonal morphological space revealed that K13 was separated from both K9 and K10 (Figure 7A). Analysis of the Euclidean distance from the mean of K9 and K10 indicated that K13 ranked 15th among the strains analyzed (Figure 7B), confirming that K13 was largely different from its parents, K9 and K10, in morphology.

Morphological changes upon crossbreeding were compared with those induced by mutation breeding using Euclidean distance (Figure 7C). We found that hybrid diploids generated with or without mutagens resulted in large morphological changes, confirming that crossbreeding of sake yeast is a good strategy to induce a variety of phenotypes.

Hybrids frequently outperform their parents, a phenomenon known as heterosis (Bernardes *et al.* 2017). In parallel, crossbreeding of genetically closely related individuals results in unexpected phenotypes not observed in the parents, a phenomenon known as inbreeding. To understand the specific genetic phenomena observed in sake yeast crossbreeding, we compared the phenotypic diversity of the progeny of the sake yeast crosses with that of the segregants from crosses with distant strains. For this purpose, we used previously published data on segregants (Nogami *et al.* 2007) from two distantly related yeast strains, laboratory yeast (BY) and wine yeast (RM). We found that the changes in Euclidean distance of the segregants in sake yeast crosses were all significantly larger than that between BY and RM at *P* < 0.01 after Bonferroni correction by Wilcoxon–Mann–Whitney *U*-test (Figure 7D). These results suggested that large morphological changes upon crossbreeding cannot be explained solely by heterosis. Moreover, we examined regression toward the mean in sake breeding. In general, hybrids tend to be closer to the average, termed “regression toward the mean” (Gamel and Axelrod 1991; Galton 1889), but this was not the case for these sake yeast strain hybrids. All of the sake hybrids had more abnormal morphology than their parents (Figure S6 in File S1), consistent with our conclusion that inbreeding caused, at least in part, the high morphological diversity of sake strains.

### Phenotypic noise of sake yeast strains

Phenotypic noise, defined as trait variability among isogenic cells sharing a common environment, is a good indicator of robustness in a cell system. If the system is robust, phenotypic noise is generally low, indicating a homogeneous cell population. In contrast, morphological heterogeneity in a cell population is largely due to impairment of cellular network hubs (Levy and Siegal 2008). Since the production of sake is optimized for a fragile two-step fermentation, every factor, including the fermenting equipment and the fermenting micro-organisms, requires a robust system. A recent study indicated that morphological robustness can be used as a good marker for breeding risk-free yeast (Tamura *et al.* 2015).

**Figure 8:**
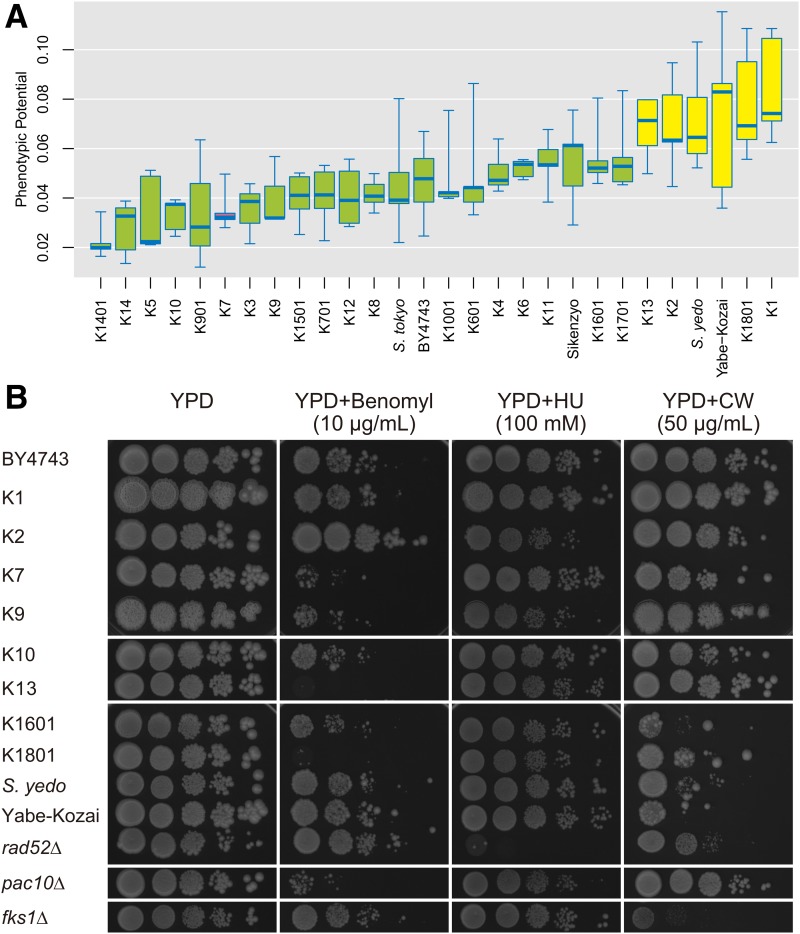
Robustness of the cellular system in sake yeast strains. (A) Distribution of phenotypic noise among the sake strains. Yellow boxes indicate the sake yeasts in which phenotypic noise was significantly higher than that of the K7 strain (*P* < 0.05, Dunnett’s test). The bottom and top of the box indicate the first and third quantiles, respectively. The band inside the box indicates the median. The bottom and top of the whiskers indicate the minimum and the maximum, respectively. (B) Sensitivity of the sake yeast strains to various robustness-disrupting stressors. Yeast strains were cultured in liquid YPD medium, and diluted serially to 1 × 10^7^, 1 × 10^6^, 1 × 10^5^, 1 × 10^4^, and 1 × 10^3^ cells/ml. 5-μl drops were spotted on plates containing benomyl (10 μg/ml), hydroxyurea (HU) (100 mM), calcofluor white (CW) (50 μg/ml), or DMSO (0.2%) as a control. Consequently, 5 × 10^4^, 5 × 10^3^, 5 × 10^2^, 5 × 10^1^, and 5 cells were plated in each spot. The plates were incubated at 25° for 5 d.

To determine the system robustness in sake yeast strains, we measured the PP as described previously (Levy and Siegal 2008; Yvert *et al.* 2013). PP is a global scalar value calculated from 220 morphological parameters that serves as an indicator of system instability. We observed variations in PP in sake yeast strains (Figure 8A) consistent with the previous finding that phenotypic noise does differ quantitatively among natural yeast isolates (Yvert *et al.* 2013). The most robust strain was K1401 (PP = 0.022 ± 0.007), while the most unstable strain was an old sake strain, K1 (PP = 0.084 ± 0.021). Compared with the standard sake yeast strain, K7, six strains (K13, K2, *S. yedo*, Yabe-Kozai, K1801, and K1) had statistically larger PPs (Dunnett’s test, *P* < 0.05); suggesting that some cellular network hubs are perturbed in these strains.

To determine which network hub is weakened in the sake strains with a larger PP, we examined the spindle assembly checkpoint (SAC), the DNA-integrity checkpoint (DIC), and cell wall integrity (CWI) activity by testing sensitivity to benomyl (Hoyt *et al.* 1991; Li and Murray 1991), hydroxyurea (Weinert *et al.* 1994; Allen *et al.* 1994), and calcofluor white (Ram and Klis 2006), respectively. In addition to our previous report on the benomyl hypersensitivity of K1801 (Tamura *et al.* 2015), we found that K13 was more sensitive to benomyl than its parents, K9 and K10 (Figure 8B). It was recently revealed that K1801 and K13 have a common chromosomal SNP in *CDC55*, responsible for the benomyl-sensitive phenotype (Goshima *et al.* 2016). K2 was slightly sensitive to 100 μg/ml hydroxyurea (Figure 8B) and *S. yedo* and Yabe-Kozai strains were sensitive to 50 μg/ml calcofluor white (Figure 8B), although their parents were not tested due to missing information of the parental strain. These findings suggest that various cellular network hubs are perturbed in some of the sake strains, creating a risk factor for robust production of sake.

## Discussion

Understanding the diversification of sake yeast along with the history of selection and breeding is important for capturing and harnessing its ability to produce sake. Our analyses indicate that the morphological diversity of sake yeast has expanded substantially compared to that of the *S*. *cerevisiae* species as a whole. Morphological profiles were associated with genotypes. We evaluated each type of breeding and found that crossbreeding resulted in more profound effects on morphology than mutation breeding. Analysis of phenotypic robustness revealed an impairment of important cellular network hubs in some of the sake yeast strains. This work provides a solid foundation for the lineage of sake yeast, understanding the origins and evolution of domesticated organisms, and proposes a new application for micro-organism breeding that will be useful in the future.

### High morphological diversity

One of the signature features of the sake yeast population is its high morphological diversity. Although the genetic diversity of sake yeast is low, its morphological diversity reached >80% of the natural *S. cerevisiae* isolates examined. Several explanations are possible for this considerable morphological diversity. First, sake yeast strains are comprised of two morphologically distinct groups, the K7 and old sake yeast groups. Since the morphological characteristics of these two groups differ, combining these populations likely results in greater morphological diversity. Second, crossbreeding likely also expands morphological diversity. We provide evidence showing that crossbreeding has profound effects on cell morphology and that the morphology of sake yeast strains changed considerably with inbreeding. The accumulation of such morphological changes during inbreeding may increase the diversity of the sake yeast strains.

Since morphological features are not selected against during breeding, morphological changes are primarily unrelated to each of the breeding objectives. Instead, morphological changes are likely accumulated accidentally in the population. Therefore, the existence of this diversity in sake yeasts allows us to use morphology as a suitable marker to evaluate each breeding step. The ideal case is to minimize the unexpected morphological changes that may occur during breeding. By monitoring high-dimensional morphological phenotypes, we obtained valuable information on the development of sake yeast.

### Morphological changes over the last century

Using PCA analysis and hierarchical clustering, we found that currently-distributed K7-lineage strains have distinct cell morphology. This serves as a useful tool to distinguish inoculated K7-lineage strains from naturally emerging unfavorable yeasts during sake brewing. Selection media containing β-alanine (Sugama *et al.* 1965) or 2,3,5-Triphenyl tetrazolium chloride (Nakamura 1998) have been used to distinguish sake yeasts from other unfavorable yeasts. Morphological examination may replace these methods if a system for monitoring yeast cell morphology is introduced in every sake brewery.

Old sake strains were morphologically different from the K7-lineage strains currently in use. We found that cells of the old sake strains are larger than the K7-lineage strains, and that the old sake strains accumulate budded cells. Based on the morphological database of nonessential deletion mutants (Ohya *et al.* 2005), impairment of 23 genes results in similar phenotypes to those observed in old sake yeast strains (large cell size and high ratio of budded cells). The gene ontology (GO) terms “cellular response to stress (GO:0033554)” and “double-strand break repair (GO:0006302)” are enriched significantly (Fisher’s exact test, *P* < 0.05 after Bonferroni correction) among these genes, suggesting that the genes required for DNA repair and the stress response are impaired in the old sake strains. It is interesting to note that the stress response genes *SWI4* and *SWI6*, whose mutants show a similar phenotype to the old strains, are involved in alcohol tolerance (Teixeira *et al.* 2009; Yoshikawa *et al.* 2009). Therefore, it is possible that a loss of function of these genes occurred in old sake strains, resulting in potential disadvantages during sake brewing. This idea is supported by the potentially deleterious mutation in *SWI6* (G280R) that was found in many old sake strains (A. Friedrich and J. Schacherer, unpublished results). An alternative possibility is that the K7-lineage strains have common loss-of-function mutations responsible for the morphological changes. The deletion of *VPS36* produced a similar morphology to the K7-lineage strains in the morphological database (Ohya *et al.* 2005) and a higher fermentation rate (D. Watanabe, unpublished results). Thus, SNPs in these genes may be responsible for the morphological phenotypes and high fermentation abilities of K7-lineage strains. Since the K7-group yeast strains produce flavored sake, it is interesting to wonder whether there is a direct relationship between morphology and fermentation properties.

### Comparison between sake and wine yeasts

Comparing sake and wine yeasts is interesting in terms of the two populations of fermenting yeasts. Sake yeasts were developed for fermentation under unusual conditions with the concerted action of filamentous fungi and yeast, resulting in a high alcohol concentration (16–20%). Sake yeast must therefore be highly alcohol tolerant. Fermentation properties at high alcohol concentrations are also important for wine yeasts, but the final concentration of alcohol in wine is lower than in sake (10–14%). An absence of organoleptic defects is required for both sake and wine yeasts, but the problematic compounds differ. Sake yeast strains with less cell lysis are preferable, because lysis produces dimethyl trisulfide (DMTS), which creates an off flavor in sake (Sasaki *et al.* 2014). Wine yeast strains to be avoided are those which compromise wine quality by producing organoleptic defects, such as volatile acidity caused by acetate (Giudici and Zambonelli 1992; Marullo *et al.* 2004), hydrogen sulfide (Giudici and Kunkee 1994; Jiranek *et al.* 1995), and phenolic compounds (Shinohara *et al.* 2000). All of the original sake yeasts were isolated from a sake brewery in Japan, while the historical progenitor of wine yeast was recently found in Mediterranean oaks (Almeida *et al.* 2015). This explains why the genetic diversity among sake yeast strains and among wine yeast strains is similarly small. The π score in sake strains is 0.9 × 10^−3^, and 1.1 × 10^−3^ in wine strains (Almeida *et al.* 2015). In this study, we found substantial phenotypic diversity in sake yeast. It will be interesting to investigate the phenotypic diversification of wine yeasts in the future.

### Risk-free sake yeast strains

We found that six sake strains have high PP. Among them, K1801 and K13 showed more sensitivity to benomyl than their parents. Since benomyl targets microtubules and activates the SAC, benomyl-sensitive K1801 and K13 strains are likely to be defective in this checkpoint, exhibiting chromosomal unstable phenotypes. Likewise, the hydroxyurea-sensitive K2 strain and the calcofluor white-sensitive *S. yedo* and Yabe-Kozai strains were likely impaired in the DIC and CWI, respectively. Given that impairment of network hubs results in high PP (Levy and Siegal 2008), our results suggest that different cellular network hubs are perturbed in these strains. It is interesting that five (K13, K2, *S. yedo*, Yabe-Kozai, and K1) of the six sake strains with higher PP are no longer commercially distributed in Japan. This may be because these strains had some disadvantages or did not have clear advantages during the sake brewing process. We propose that assessing the PP will be useful for developing risk-free yeast strains.

### High-dimensional phenotyping during inbreeding

Inbreeding dramatically reduces the viability of offspring due to increasing genetic homozygosity. As a result, measuring reductions in viability is crucial in domesticated farm animals, including Holsteins (Sun *et al.* 2013), horses (Schubert *et al.* 2014), and pigs (Herrero-Medrano *et al.* 2014); as well as in agricultural crops, including rice (Doi *et al.* 2008), wheat (Whitford *et al.* 2013), and corn (Schneider *et al.* 2016). Rather than a reduction in viability, we observed a considerably high degree of morphological changes during crossbreeding of closely related sake yeast strains. Our analysis suggested that inbreeding sake yeast strains caused, at least in part, the high morphological diversity of yeast cells. Therefore, we propose that high-dimensional morphological phenotyping can be used to monitor the risks of homozygosity. It is of interest to test whether such expansion of morphological diversity in the population is also frequently observed in other inbreeding processes.

### Application of high-dimensional phenotyping during breeding and brewing processes

We propose that high-dimensional morphological phenotyping is applicable to several aspects of the breeding and brewing of micro-organisms. First, breeding organisms can be classified based on morphological phenotypes. It is particularly attractive when morphological profiles are associated with genotypes, as is the case for the sake yeast strains. Classification based on cell morphology using image processing systems and statistical analyses may provide opportunities to predict the origin of a strain geographically or ecologically. In a more practical sense, during brewing, classification provides information to distinguish invading strains from the inoculated strain. Second, morphological features can be used to select for desired or undesired traits for fermentation. A significant increase of PP, or higher morphological variability, suggests the acquisition of a risk factor for robust fermentation. Therefore, keeping PP scores low is supposed to be preferable during breeding. Finding any correlation between morphology and desired traits would present a direct strategy for breeding. In this situation, we assume that the morphological changes and the desired traits are caused by the same driver mutation. Third, high-dimensional phenotyping is also useful to check for the existence of off-target mutations accidentally incorporated during breeding. When a lineage map is known, the degree of morphological changes between parent and progeny can be evaluated individually as Euclidian distances, just like our analyses on sake yeast strains. We showed that the use of mutagens resulted in substantial morphological alterations, possibly due to the accumulation of off-target mutations. One potential breeding strategy is to exclude undesirable segregants with large amounts of unexpected morphological changes. We propose that the undesirable segregants can be excluded by morphological screening. Finally, it should be emphasized that combined with other omics studies, including metabolomics (Mimura *et al.* 2014; Takahashi *et al.* 2017) and transcriptomics (Hirasawa *et al.* 2010), high-dimensional phenotyping of sake yeast strains has a wider application. Correlation analyses among omics data may give us fundamental knowledge as well as possible screening strategies for breeding. High-dimensional phenotyping generates new, and significantly more, data than ever before. It requires novel data management and new statistical tools for extracting biologically meaningful information. This study provides methods for improving the understanding of breeding organisms and obtaining new knowledge for efficiently breeding micro-organism varieties in the future.

## Supplementary Material

Supplemental material is available online at www.g3journal.org/lookup/suppl/doi:10.1534/g3.117.044099/-/DC1.

## References

[bib1] Akao T., 2014 New perspectives of genetic variability of sake yeast strains in microevolution and breeding. Kagaku To Seibutsu 52: 223–232.

[bib2] Akao T., 2015 Genomics of the sake yeasts, pp. 117–126 in *The Frontier of Fermented Foods*, edited by Kitamoto K. CMC Publishing, Tokyo.

[bib3] Akao T. Yashiro I. Hosoyama A. Kitagaki H. Horikawa H., 2011 Whole-genome sequencing of sake yeast *Saccharomyces cerevisiae* Kyokai no. 7. DNA Res. 18: 423–434. 21900213 10.1093/dnares/dsr029 PMC3223075

[bib4] Akiyama H., 2010 *Sake: The Essence of 2000 Years of Japanese Wisdom Gained from Brewing Alcoholic Beverages from Rice* Brewing Society of Japan, Tokyo, Japan.

[bib5] Allen J. B. Zhou Z. Siede W. Friedberg E. C. Elledge S. J., 1994 The SAD1/RAD53 protein kinase controls multiple checkpoints and DNA damage-induced transcription in yeast. Genes Dev. 8: 2401–2415. 7958905 10.1101/gad.8.20.2401

[bib6] Almeida P. Barbosa R. Zalar P. Imanishi Y. Shimizu K., 2015 A population genomics insight into the Mediterranean origins of wine yeast domestication. Mol. Ecol. 24: 5412–5427. 26248006 10.1111/mec.13341

[bib7] Amos W. Harwood J., 1998 Factors affecting levels of genetic diversity in natural populations. Philos. Trans. R. Soc. Lond. B Biol. Sci. 353: 177–186. 9533122 10.1098/rstb.1998.0200 PMC1692205

[bib8] Bernardes J. P. Stelkens R. B. Greig D., 2017 Heterosis in hybrids within and between yeast species. J. Evol. Biol. 30: 538–548. 27933674 10.1111/jeb.13023

[bib9] Bourguiba H. Audergon J. M. Krichen L. Trifi-Farah N. Mamouni A., 2012 Loss of genetic diversity as a signature of apricot domestication and diffusion into the Mediterranean Basin. BMC Plant Biol. 12: 49. 22510209 10.1186/1471-2229-12-49 PMC3511222

[bib10] Casey G. P. Ingledew W. M., 1986 Ethanol tolerance in yeasts. Crit. Rev. Microbiol. 13: 219–280. 3533426 10.3109/10408418609108739

[bib11] Cromie G. A. Hyma K. E. Ludlow C. L. Garmendia-Torres C. Gilbert T. L., 2013 Genomic sequence diversity and population structure of *Saccharomyces cerevisiae* assessed by RAD-seq. G3 (Bethesda) 3: 2163–2171. 24122055 10.1534/g3.113.007492 PMC3852379

[bib12] Crow J. F., 2010 Wright and Fisher on inbreeding and random drift. Genetics 184: 609–611. 20332416 10.1534/genetics.109.110023 PMC2845331

[bib13] Deza M. Deza E., 2009 *Encyclopedia of Distances* Springer Verlag, Berlin.

[bib14] Doi K. Yasui H. Yoshimura A., 2008 Genetic variation in rice. Curr. Opin. Plant Biol. 11: 144–148. 18316240 10.1016/j.pbi.2008.01.008

[bib15] Galton F., 1889 *Natural Inheritance* Macmillan, London.

[bib16] Gamel J. W. Axelrod D. E., 1991 Inheritance and regression toward the mean in heterogeneous cell populations. Cell Prolif. 24: 281–292. 2039804 10.1111/j.1365-2184.1991.tb01157.x

[bib17] Giudici P. Kunkee R. E., 1994 The effect of nitrogen deficiency and sulfur-containing amino-acids on the reduction of sulfate to hydrogen-sulfide by wine yeasts. Am. J. Enol. Vitic. 45: 107–112.

[bib18] Giudici P. Zambonelli C., 1992 Biometric and genetic-study on acetic-acid production for breeding of wine yeast. Am. J. Enol. Vitic. 43: 370–374.

[bib19] Goshima T. Nakamura R. Kume K. Okada H. Ichikawa E., 2016 Identification of a mutation causing a defective spindle assembly checkpoint in high ethyl caproate-producing sake yeast strain K1801. Biosci. Biotechnol. Biochem. 80: 1657–1662. 27191586 10.1080/09168451.2016.1184963

[bib20] Hara S., 1978 Development of ethanol-resistant sake yeast. Journal of the Brewing Society of Japan 73: 701–703.

[bib21] Herrero-Medrano J. M. Megens H. J. Groenen M. A. Bosse M. Perez-Enciso M., 2014 Whole-genome sequence analysis reveals differences in population management and selection of European low-input pig breeds. BMC Genomics 15: 601. 25030608 10.1186/1471-2164-15-601 PMC4117957

[bib22] Hirasawa T. Furusawa C. Shimizu H., 2010 *Saccharomyces cerevisiae* and DNA microarray analyses: what did we learn from it for a better understanding and exploitation of yeast biotechnology? Appl. Microbiol. Biotechnol. 87: 391–400. 20414652 10.1007/s00253-010-2582-7

[bib23] Hothorn T. Bretz F. Westfall P., 2008 Simultaneous inference in general parametric models. Biom. J. 50: 346–363. 18481363 10.1002/bimj.200810425

[bib24] Hoyt M. A. Totis L. Roberts B. T., 1991 *S. cerevisiae* genes required for cell cycle arrest in response to loss of microtubule function. Cell 66: 507–517. 1651171 10.1016/0092-8674(81)90014-3

[bib25] Ichikawa E. Hosokawa N. Hata Y. Abe Y. Suginami K., 1991 Breeding of a sake yeast with improved ethyl caproate productivity. Agric. Biol. Chem. 55: 2153–2154.

[bib26] Jiranek V. Langridge P. Henschke P. A., 1995 Validation of bismuth-containing indicator media for predicting H_2_S-producing potential of *Saccharomyces cerevisiae* wine yeasts under enological conditions. Am. J. Enol. Vitic. 46: 269–273.

[bib27] Jung P. P. Sigwalt A. Ohnuki S. de Montigny J. Ohya Y., 2016 Large-scale survey of intraspecific fitness and cell morphology variation in a protoploid yeast species. G3 (Bethesda) 6: 1063–1071. 26888866 10.1534/g3.115.026682 PMC4825641

[bib28] Kanauchi M., 2013 *SAKE Alcoholic Beverage Production in Japanese Food Industry* INTECH Open Access Publisher, Rijeka, Croatia.

[bib29] Kitagaki H. Kitamoto K., 2013 Breeding research on sake yeasts in Japan: history, recent technological advances, and future perspectives. Annu. Rev. Food Sci. Technol. 4: 215–235. 23464572 10.1146/annurev-food-030212-182545

[bib30] Levy S. F. Siegal M. L., 2008 Network hubs buffer environmental variation in *Saccharomyces cerevisiae*. PLoS Biol. 6: e264. 18986213 10.1371/journal.pbio.0060264 PMC2577700

[bib31] Li R. Murray A. W., 1991 Feedback control of mitosis in budding yeast. Cell 66: 519–531. 1651172 10.1016/0092-8674(81)90015-5

[bib32] Liti G. Carter D. M. Moses A. M. Warringer J. Parts L., 2009 Population genomics of domestic and wild yeasts. Nature 458: 337–341. 19212322 10.1038/nature07743 PMC2659681

[bib33] Marullo P. Bely M. Masneuf-Pomarede I. Aigle M. Dubourdieu D., 2004 Inheritable nature of enological quantitative traits is demonstrated by meiotic segregation of industrial wine yeast strains. FEMS Yeast Res. 4: 711–719. 15093774 10.1016/j.femsyr.2004.01.006

[bib34] Mimura N. Isogai A. Iwashita K. Bamba T. Fukusaki E., 2014 Gas chromatography/mass spectrometry based component profiling and quality prediction for Japanese sake. J. Biosci. Bioeng. 118: 406–414. 25060729 10.1016/j.jbiosc.2014.04.006

[bib35] Nakamura T., 1998 Management of microorganisms. Journal of the Brewing Society of Japan 93: 586–593.

[bib36] Nakazawa R., 1909 Zwei Saccharomyceten aus Sakehefe. Central. f. Bakt. Abt. II 22: 529–540.

[bib37] Nogami S. Ohya Y. Yvert G., 2007 Genetic complexity and quantitative trait loci mapping of yeast morphological traits. PLoS Genet. 3: e31. 17319748 10.1371/journal.pgen.0030031 PMC1802830

[bib38] Ogawa Y. Nitta A. Uchiyama H. Imamura T. Shimoi H., 2000 Tolerance mechanism of the ethanol-tolerant mutant of sake yeast. J. Biosci. Bioeng. 90: 313–320. 16232862 10.1016/s1389-1723(00)80087-0

[bib39] Ohnuki S. Kobayashi T. Ogawa H. Kozone I. Ueda J. Y., 2012 Analysis of the biological activity of a novel 24-membered macrolide JBIR-19 in *Saccharomyces cerevisiae* by the morphological imaging program CalMorph. FEMS Yeast Res. 12: 293–304. 22129199 10.1111/j.1567-1364.2011.00770.x

[bib40] Ohnuki S. Enomoto K. Yoshimoto H. Ohya Y., 2014 Dynamic changes in brewing yeast cells in culture revealed by statistical analyses of yeast morphological data. J. Biosci. Bioeng. 117: 278–284. 24012106 10.1016/j.jbiosc.2013.08.005

[bib41] Ohya Y. Sese J. Yukawa M. Sano F. Nakatani Y., 2005 High-dimensional and large-scale phenotyping of yeast mutants. Proc. Natl. Acad. Sci. USA 102: 19015–19020. 16365294 10.1073/pnas.0509436102 PMC1316885

[bib42] Ohya Y. Kimori Y. Okada H. Ohnuki S., 2015 Single-cell phenomics in budding yeast. Mol. Biol. Cell 26: 3920–3925. 26543200 10.1091/mbc.E15-07-0466 PMC4710224

[bib43] Ouchi K. Akiyama H., 1971 Non-foaming mutants of sake yeasts - selection by cell agglutination method and by froth flotation method. Agric. Biol. Chem. 35: 1024–1032.

[bib44] Ouchi K. Nunokawa Y., 1973 Non-foaming mutants of sake yeast - their physicochemical characteristics. J. Ferment. Technol. 51: 85–95.

[bib45] Paradis E. Claude J. Strimmer K., 2004 APE: analyses of phylogenetics and evolution in R language. Bioinformatics 20: 289–290. 14734327 10.1093/bioinformatics/btg412

[bib46] Ram A. F. Klis F. M., 2006 Identification of fungal cell wall mutants using susceptibility assays based on Calcofluor white and Congo red. Nat. Protoc. 1: 2253–2256. 17406464 10.1038/nprot.2006.397

[bib47] Sasaki K. Nishibori N. Kanai M. Isogai A. Yamada O., 2014 Statistical analysis of sake-preparation conditions and dimethyl trisulfide formation. J. Biosci. Bioeng. 118: 166–171. 24525110 10.1016/j.jbiosc.2014.01.005

[bib48] Schacherer J. Shapiro J. A. Ruderfer D. M. Kruglyak L., 2009 Comprehensive polymorphism survey elucidates population structure of *Saccharomyces cerevisiae*. Nature 458: 342–345. 19212320 10.1038/nature07670 PMC2782482

[bib49] Schneider K. L. Xie Z. Wolfgruber T. K. Presting G. G., 2016 Inbreeding drives maize centromere evolution. Proc. Natl. Acad. Sci. USA 113: E987–E996. 26858403 10.1073/pnas.1522008113 PMC4776452

[bib50] Schubert M. Jonsson H. Chang D. Der Sarkissian C. Ermini L., 2014 Prehistoric genomes reveal the genetic foundation and cost of horse domestication. Proc. Natl. Acad. Sci. USA 111: E5661–E5669. 25512547 10.1073/pnas.1416991111 PMC4284583

[bib51] Shimoi H. Sakamoto K. Okuda M. Atthi R. Iwashita K., 2002 The *AWA1* gene is required for the foam-forming phenotype and cell surface hydrophobicity of sake yeast. Appl. Environ. Microbiol. 68: 2018–2025. 11916725 10.1128/AEM.68.4.2018-2025.2002 PMC123892

[bib52] Shinohara T. Kubodera S. Yanagida F., 2000 Distribution of phenolic yeasts and production of phenolic off-flavors in wine fermentation. J. Biosci. Bioeng. 90: 90–97. 16232824 10.1016/s1389-1723(00)80040-7

[bib53] Shiroma S. Jayakody L. N. Horie K. Okamoto K. Kitagaki H., 2014 Enhancement of ethanol fermentation in *Saccharomyces cerevisiae* sake yeast by disrupting mitophagy function. Appl. Environ. Microbiol. 80: 1002–1012. 24271183 10.1128/AEM.03130-13 PMC3911210

[bib54] Stasinopoulos D. M. Rigby R. A., 2007 Generalized additive models for location scale and shape (GAMLSS) in R. J. Stat. Softw. 23. DOI: 10.18637/jss.v023.i07.

[bib55] Strope P. K. Skelly D. A. Kozmin S. G. Mahadevan G. Stone E. A., 2015 The 100-genomes strains, an *S. cerevisiae* resource that illuminates its natural phenotypic and genotypic variation and emergence as an opportunistic pathogen. Genome Res. 25: 762–774. 25840857 10.1101/gr.185538.114 PMC4417123

[bib56] Sugama S. Yamakawa K. Kataoka G. Yamamura K. Noshiro K., 1965 Study on sake yeast in sake breweries (2). Journal of the Brewing Society of Japan 60: 453–456.

[bib57] Sun C. VanRaden P. M. O’Connell J. R. Weigel K. A. Gianola D., 2013 Mating programs including genomic relationships and dominance effects. J. Dairy Sci. 96: 8014–8023. 24119810 10.3168/jds.2013-6969

[bib58] Suzuki R. Shimodaira H., 2006 Pvclust: an R package for assessing the uncertainty in hierarchical clustering. Bioinformatics 22: 1540–1542. 16595560 10.1093/bioinformatics/btl117

[bib59] Takahashi A. Kono S. Wada A. Oshima S. Abe K., 2017 Reduced brain activity in female patients with non-alcoholic fatty liver disease as measured by near-infrared spectroscopy. PLoS One 12: e0174169. 28376101 10.1371/journal.pone.0174169 PMC5380307

[bib60] Tamura H. Okada H. Kume K. Koyano T. Goshima T., 2015 Isolation of a spontaneous cerulenin-resistant sake yeast with both high ethyl caproate-producing ability and normal checkpoint integrity. Biosci. Biotechnol. Biochem. 79: 1191–1199. 25787154 10.1080/09168451.2015.1020756

[bib61] Teixeira M. C. Raposo L. R. Mira N. P. Lourenco A. B. Sa-Correia I., 2009 Genome-wide identification of *Saccharomyces cerevisiae* genes required for maximal tolerance to ethanol. Appl. Environ. Microbiol. 75: 5761–5772. 19633105 10.1128/AEM.00845-09 PMC2747848

[bib62] Tsukahara T., 1962 Taxonomic study of sake yeast (4). Journal of the Brewing Society of Japan 57: 117–123.

[bib63] Watanabe D. Nogami S. Ohya Y. Kanno Y. Zhou Y., 2011 Ethanol fermentation driven by elevated expression of the G1 cyclin gene *CLN3* in sake yeast. J. Biosci. Bioeng. 112: 577–582. 21906996 10.1016/j.jbiosc.2011.08.010

[bib64] Weinert T. A. Kiser G. L. Hartwell L. H., 1994 Mitotic checkpoint genes in budding yeast and the dependence of mitosis on DNA replication and repair. Genes Dev. 8: 652–665. 7926756 10.1101/gad.8.6.652

[bib65] Whitford R. Fleury D. Reif J. C. Garcia M. Okada T., 2013 Hybrid breeding in wheat: technologies to improve hybrid wheat seed production. J. Exp. Bot. 64: 5411–5428. 24179097 10.1093/jxb/ert333

[bib66] Yabe K., 1897 On the origin of sake yeast (*Sacch. sake*). *Coll. of Agric. Tokyo*. Bull. 3: 211–224.

[bib67] Yang M. Ohnuki S. Ohya Y., 2014 Unveiling nonessential gene deletions that confer significant morphological phenotypes beyond natural yeast strains. BMC Genomics 15: 932. 25344683 10.1186/1471-2164-15-932 PMC4221665

[bib68] Yoshikawa K. Tanaka T. Furusawa C. Nagahisa K. Hirasawa T., 2009 Comprehensive phenotypic analysis for identification of genes affecting growth under ethanol stress in *Saccharomyces cerevisiae*. FEMS Yeast Res. 9: 32–44. 19054128 10.1111/j.1567-1364.2008.00456.x

[bib69] Yvert G. Ohnuki S. Nogami S. Imanaga Y. Fehrmann S., 2013 Single-cell phenomics reveals intra-species variation of phenotypic noise in yeast. BMC Syst. Biol. 7: 54. 23822767 10.1186/1752-0509-7-54 PMC3711934

[bib70] Zeileis A. Hothorn T., 2002 Diagnostic checking in regression relationships. R News 2: 4.

