# Supplementary material for: Phenotypic Diagnosis of Lineage and Differentiation During Sake Yeast Breeding
Source: G3 (Bethesda). 2017 Jun 22;7(8):2807–20. doi: 10.1534/g3.117.044099 (PMC5555484; doi:10.1534/g3.117.044099)
Supplement: Supplementary file 1 [file 2807FileS1.pdf]

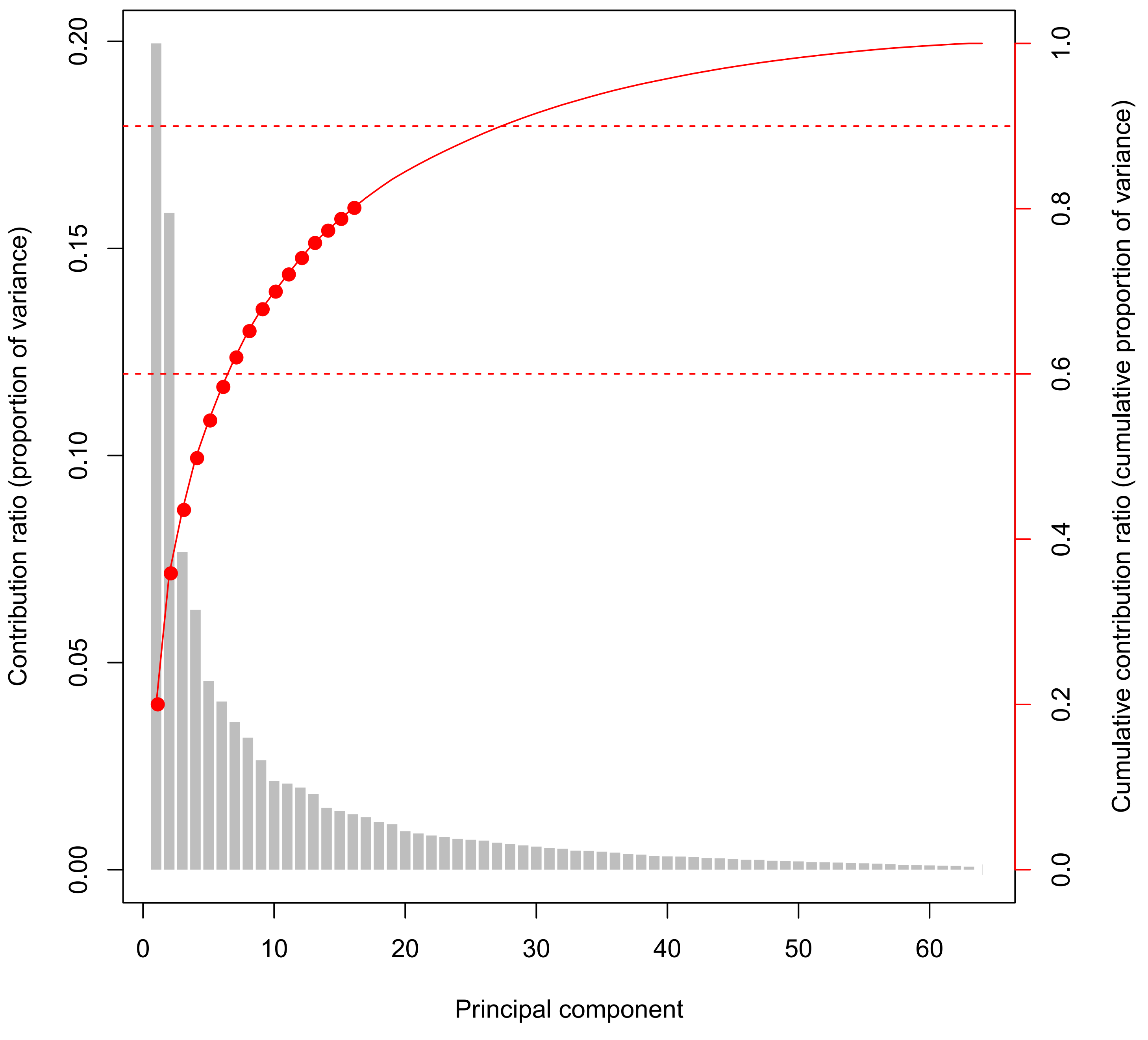

Figure S1. Cumulative contribution ratios of PCA for 64 strains.
Gray bars indicate the proportion of variance (left axis) explained by the PCs. Red circles indicate the cumulative proportion of variance (right axis) explained by the PCs. The horizontal dashed red lines indicate 60% and 90% of the cumulative proportion of variance.

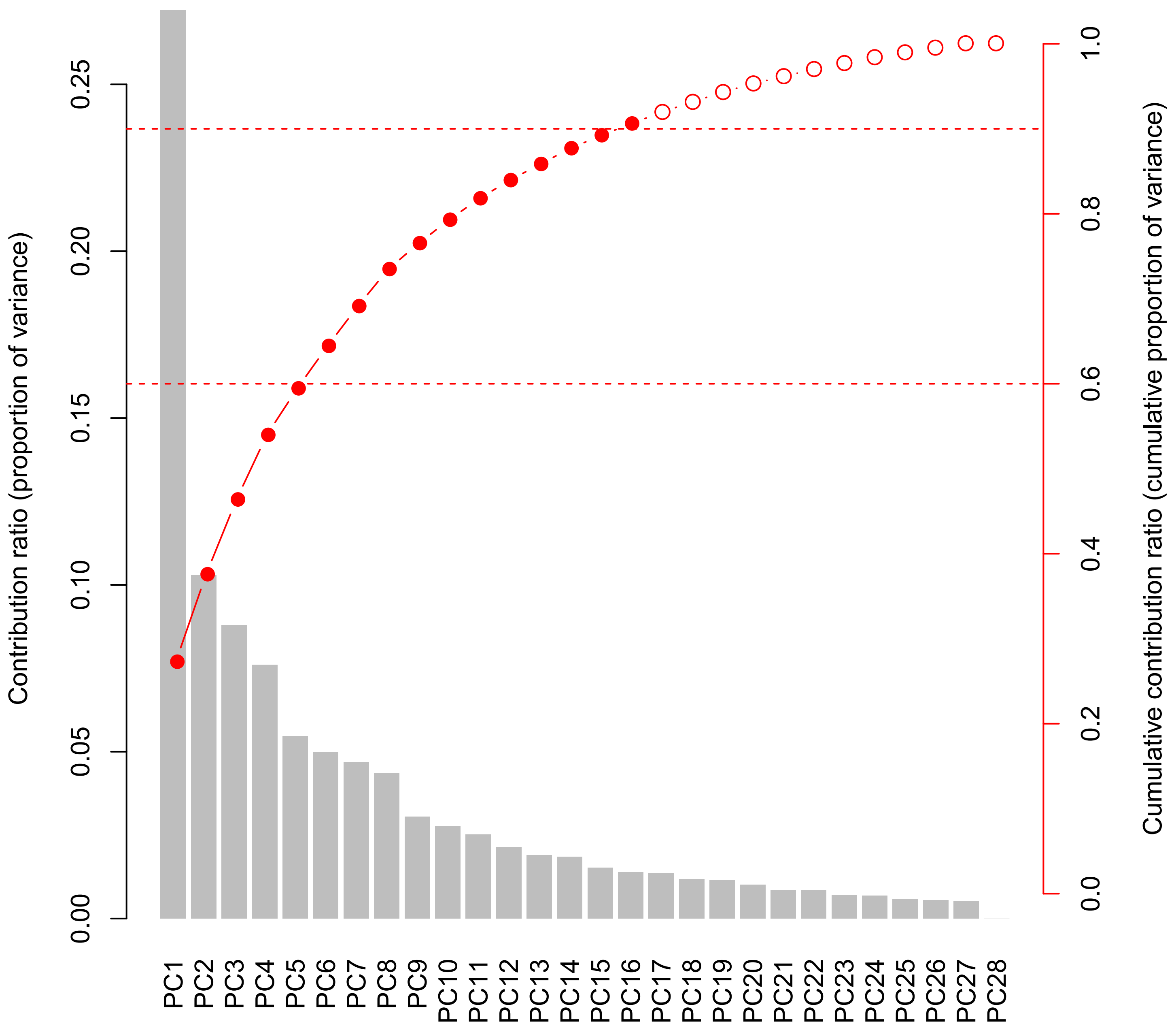

Figure S2. Cumulative contribution ratios of PCA for 27 sake yeast strains and BY4743.
Gray bars indicate the proportion of variance (left axis) explained by the PCs. Red circles indicate the cumulative proportion of variance (right axis) explained by the PCs. The horizontal dashed red lines indicate 60% and 90% of the cumulative proportion of variance.

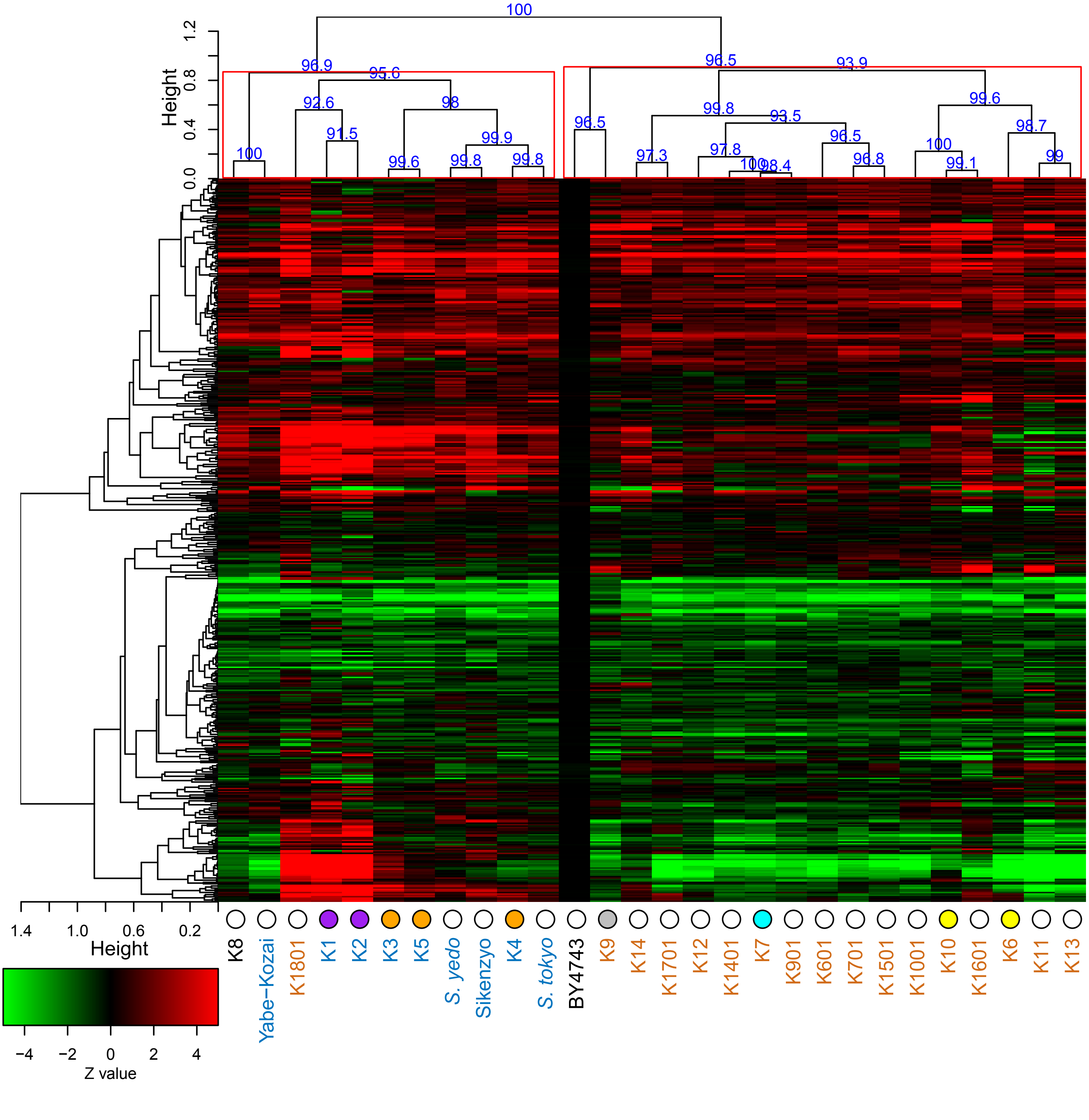

Figure S3. Heat map of morphological phenotypes in 27 sake yeast strains and BY4743.
Cluster dendrograms at the left side and top of the heat map were constructed from average linkages of correlation coefficients calculated from PC scores of 7 and 6 PCs (CCR = 90% and 60%, respectively) from PCA of 501 traits and 28 strains, respectively. Blue numbers at each cluster in the top panel indicate AU p-values calculated based on multi-scale bootstrap clustering (see Materials and Methods). Red rectangles in the top panel represent significant groups detected at AU p-values > 0.95. Green, red, and black squares in the heat map indicate Z values that were lower than, greater than, and equal to BY4743, respectively. Colors of circles and strain names are the same as in Fig. 1A and Fig. 3B, respectively.

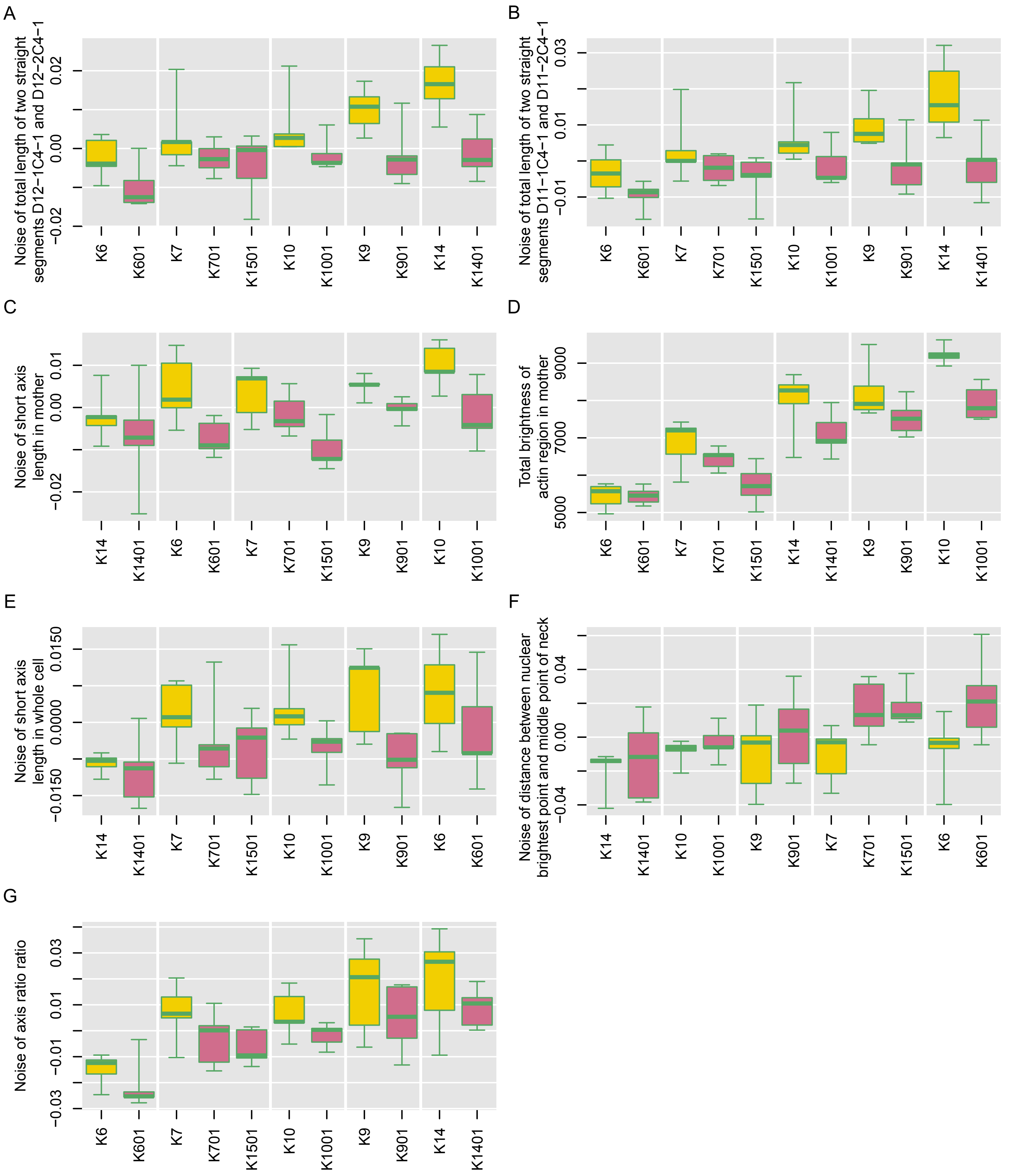

Figure S4. Morphological features of *awa* mutants.
A) DCV186_C, noise of the total length from the middle point of the neck to the respective points on the cell periphery through the brightest point of the nucleus in the mother and bud at M phase. B) DCV185_C, noise of the total length from the middle point of the neck to the respective points on the cell periphery through the center of gravity of the nucleus in the mother and bud at M phase. C) CCV104_C, noise of the short axis length in the mother at M phase. D) A8-1_A1B, total brightness of the actin region in the mother at S/G2 phase. E) CCV104_A, noise of short axis length in whole cells at G1 phase. F) DCV132_A1B, noise of distance between brightest point of nucleus and middle point of neck. G) CCV116_A1B, noise of axis ratio of bud to axis ratio of mother at G2/S. Yellow and red boxes indicate the parental strain and the *awa* mutant, respectively.

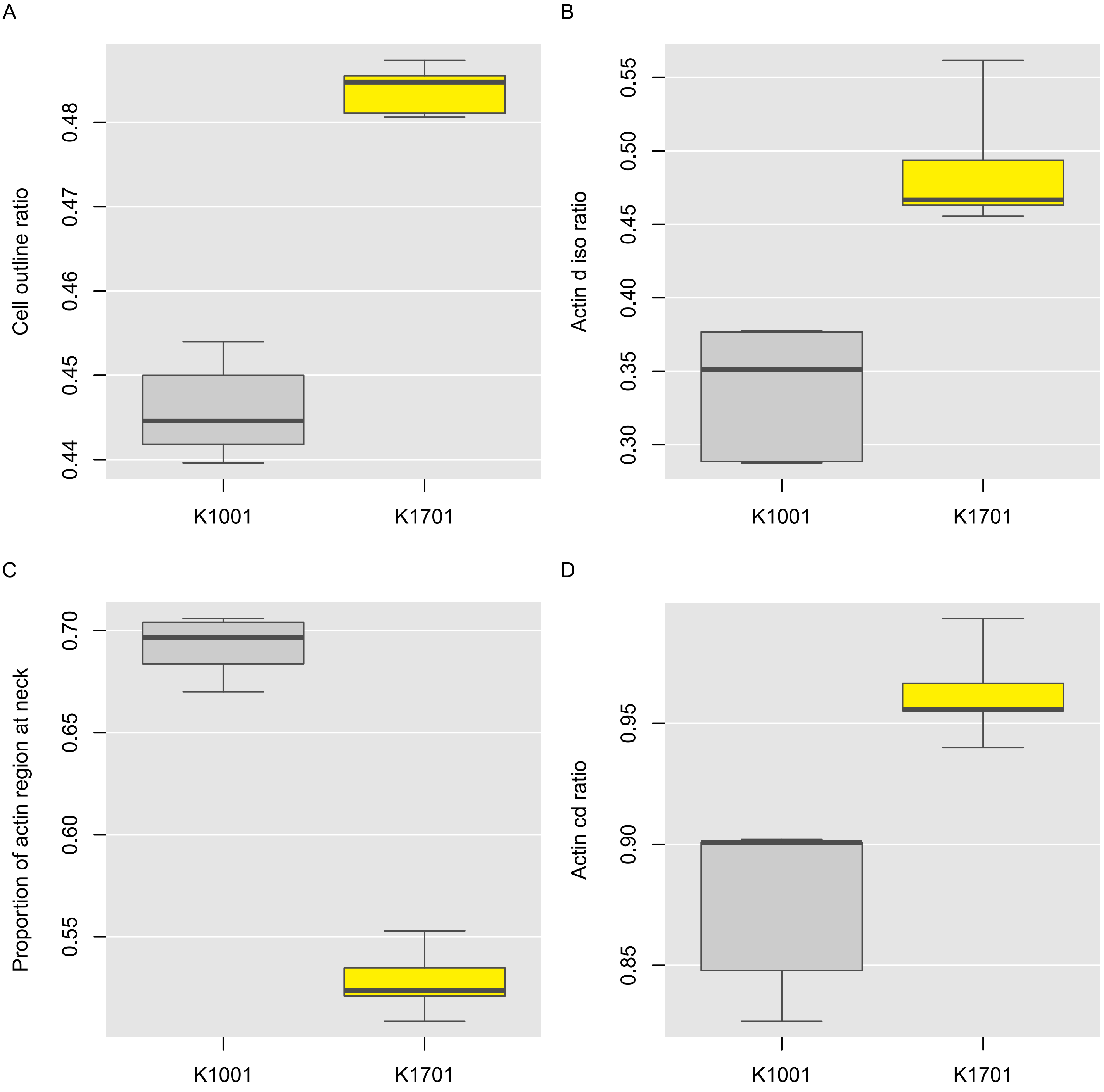

Figure S5. Representative morphological differences between K1001 and K1701.
A) C117_A1B, cell outline ratio at S/G2 phase. B) A108_A1B, ratio of cells with actin patches delocalized at bud at S/G2 phase. C) A9_A1B, proportion of actin region at neck at S/G2. D) A112_A1B, ratio of cells with actin patch at bud at G1 S/G2 phase. Four traits are represented from among the 26 traits listed in Table S4.

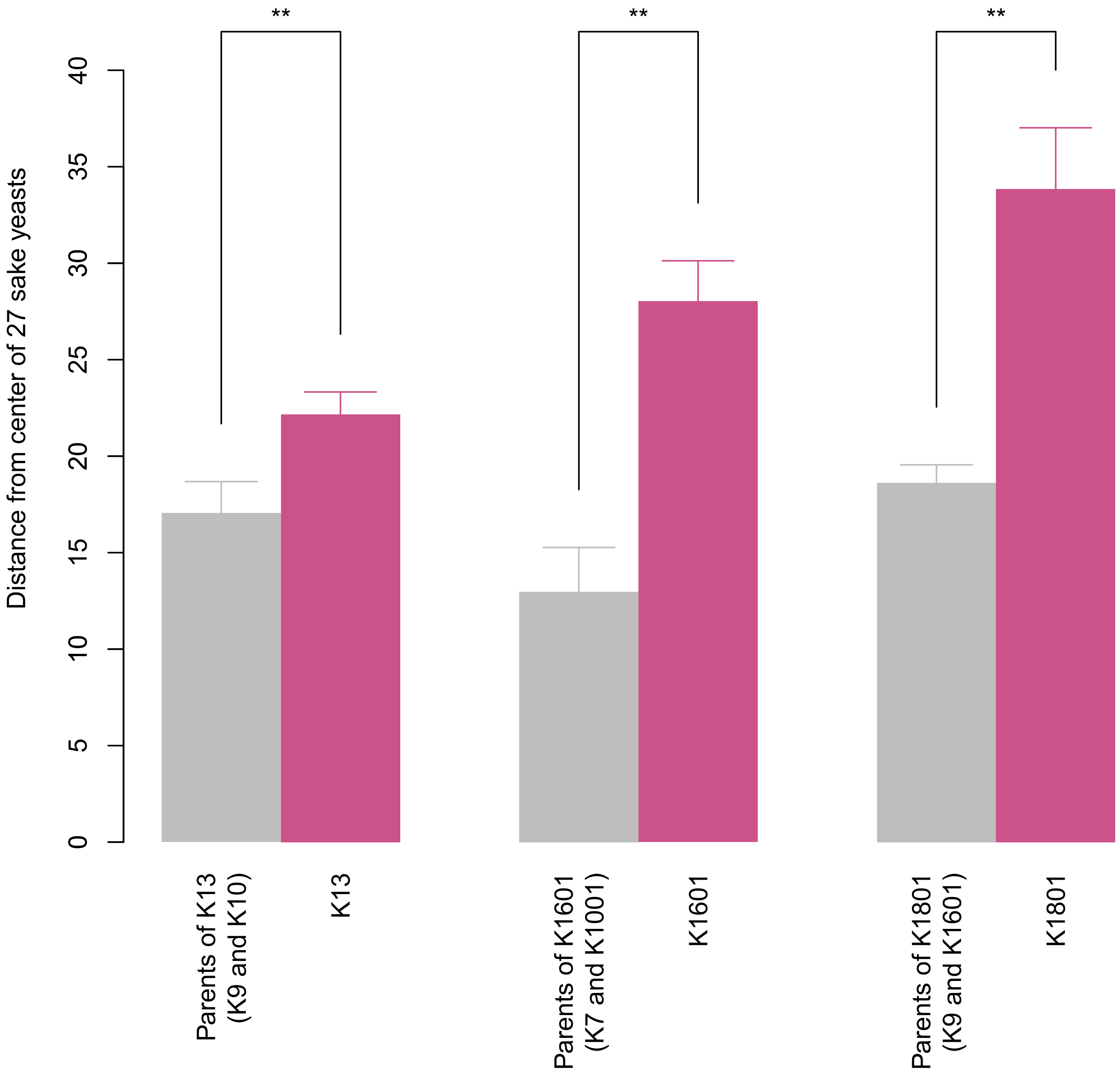

Figure S6. Morphological difference of the parents and the progeny from the average of the 27 sake yeast strains. Euclidean distance from the average of the 27 sake yeast strains was calculated from the PC scores of 16 PCs (CCR = 90%). Gray and magenta bars indicate the parents (K9 and K10, K7 and K1001, and K9 and K1601) and the progeny (K13, K1601, and K1801) of the sake yeasts, respectively. Asterisks (**) indicate $p < 0.01$ by Wilcoxon-Mann-Whitney *U* test.
